# Comparative proteomic analysis provides new insight into differential transmission of two begomoviruses by a whitefly

**DOI:** 10.1186/s12985-019-1138-4

**Published:** 2019-03-11

**Authors:** Jing Zhao, Yao Chi, Xin-Jia Zhang, Teng Lei, Xiao-Wei Wang, Shu-Sheng Liu

**Affiliations:** Ministry of Agriculture Key Laboratory of Molecular Biology of Crop Pathogens and Insects, Institute of Insect Sciences, Zhejiang University, Hangzhou, 310058 People’s Republic of China

**Keywords:** Whitefly, Begomovirus, Virus transmission, iTRAQ-based proteomics

## Abstract

**Background:**

Viruses in the genus *Begomovirus* (Family *Geminiviridae*) include many important economic plant viruses transmitted by whiteflies of the *Bemisia tabaci* species complex. In general, different begomoviruses may be acquired and transmitted by the same whitefly species with different efficiencies. For example, the species Mediterranean (MED) in this whitefly species complex transmits tomato yellow leaf curl virus (TYLCV) at a higher efficiency than papaya leaf curl China virus (PaLCuCNV). However, the proteomic responses of whitefly to the infection of different begomoviruses remain largely unknown.

**Methods:**

We used iTRAQ-based proteomics coupled with RT-qPCR to investigate and compare responses of the MED whitefly to the infection of TYLCV and PaLCuCNV.

**Results:**

Totally, 259, 395 and 74 differently expressed proteins (DEPs) were identified in the comparisons of TYLCV-infected vs. un-infected, PaLCuCNV-infected vs. un-infected, and TYLCV-infected vs. PaLCuCNV-infected whiteflies, respectively. These proteins appear associated with catabolic process, metabolic process, transport, defense response, cell cycle, and receptor. The comparisons of TYLCV-infected vs. un-infected and PaLCuCNV-infected vs. un-infected shared some similar DEPs, indicating possible involvement of laminin subunit alpha, dystroglycan, integrin alpha-PS2 and cuticle proteins in viral transport as well as the role of putative defense proteins 3 and PITH in anti-viral response. However, 20S proteasome subunits associated with regulation of virus degradation and accumulation were up-regulated in PaLCuCNV-infected but not in TYLCV-infected whiteflies, which may be related to the constraints of PaLCuCNV accumulation in MED.

**Conclusions:**

These findings provide valuable clues for unravelling the roles of some whitefly proteins in begomovirus transmission.

**Electronic supplementary material:**

The online version of this article (10.1186/s12985-019-1138-4) contains supplementary material, which is available to authorized users.

## Background

Plant diseases caused by begomoviruses have been major constraints to the production of many economic crops such as tomato and cotton [1]. Begomoviruses are a group of single-stranded circular DNA viruses with twinned particles, and their genetic structure is bipartite or monopartite [2]. So far, more than 388 species have been described in the genus *Begomovirus* (Family *Geminiviridae*) [3], which in general are transmitted by whiteflies in the *Bemisia tabaci* species complex in a persistent, circulative manner [4]. For begomoviruses, once orally acquired by whitefly, they follow the path of head-midgut-haemolymph-primary salivary gland inside the vector [5–7]. The coat protein is the only known viral structural protein in determining begomovirus transmission characteristics [8]. In the whitefly, two organs including midgut and primary salivary gland have been identified as barriers in the circulative journey of begomovirus in the vector body [9–11]. A few proteins have been investigated for their roles in begomovirus transmission. Two whitefly proteins, cyclophilin B and a midgut protein, seem to have roles in assisting begomovirus transmission [12, 13]; while another two whitefly proteins, heat shock protein 70 and Knottin-1, seem to negatively affect begomovirus transmission by whitefly [14, 15]. In addition, a peptidoglycan recognition protein and an antimicrobial peptide have been reported to be involved in whitefly-begomovirus interaction [16, 17].

Tomato yellow leaf curl virus (TYLCV) is a monopartite begomovirus without satellite DNA and is one of the most economically important begomoviruses all over the world [1, 18, 19]. Papaya leaf curl China virus (PaLCuCNV) is another monopartite begomovirus without satellite DNA, indigenous to China [20]. Both TYLCV and PaLCuCNV can be transmitted by a globally important species of whitefly, provisionally named as Mediterranean (MED), in the *B. tabaci* complex [21–24]. Previous studies showed that TYLCV can be more efficiently transmitted by MED whitefly than PaLCuCNV [11, 23, 24]. It seems common that different begomoviruses can be acquired and transmitted by the same whitefly species at different efficiencies, and different whitefly species vary in their capacity in acquiring and transmitting a given begomovirus [9, 23–25]. However, up to now, little is yet known about the molecular mechanisms underlying these differences. One way to gain understanding of the molecular mechanisms is to compare the responses of a given whitefly species to different begomoviruses which are transmitted by the whitefly with varied efficiencies, for example MED to TYLCV and PaLCuCNV.

In the past decade, transcriptomics have been used to analyze the interactions between begomoviruses and whiteflies [26–29]. However, the differently expressed proteins (DEPs) at translational level can better reflect the physiological changes induced by begomoviruses infection. Isobaric tags for relative and absolute quantification (iTRAQ)-based quantitative proteomic approach is a popular methodology in life science, which has been used to investigate the interactions of viruses with various vectors/hosts [30–34].

In this study, we collected un-infected, TYLCV-infected and PaLCuCNV-infected whiteflies respectively, and then used iTRAQ-based quantitative proteomic analysis to elucidate the interactions underlying different combinations of whitefly and begomovirus. Three comparisons were made including TYLCV-infected vs. un-infected, PaLCuCNV-infected vs. un-infected, and TYLCV-infected vs. PaLCuCNV-infected. Our objectives were to provide new visions on the interactions underlying begomovirus transmission by whiteflies and stimulate investigation on these interactions at the proteome level.

## Methods

### Insects, plants and viruses

The species of whitefly named as MED (mtCOI GenBank accession code: GQ371165) and two tomato cultivars, *Solanum lycopersicum* cv. Hezuo 903 and cv. Zheza 502, were used for experiments. Clones of TYLCV SH2 (GenBank accession number: AM282874) and PaLCuCNV isolate HeNZM1 (GenBank accession number: FN256260) were obtained from the Institute of Biotechnology, Zhejiang University.

### Preparation of whitefly samples

A culture of MED whitefly was reared on un-infected tomato plants (*S. lycopersicum* cv. Hezuo 903) in insect proof cages. For the experiments, un-infected tomato plants of both Hezuo 903 and Zheza 502 were cultivated to 7–8 true leaf stage when used. For preparation of TYLCV-infected and PaLCuCNV-infected plants, tomato of Hezuo 903 were first cultivated to 3–4 true leaf stage when virus inoculation was conducted, and then the virus-inoculated plants were further cultivated to 7–8 true leaf stage when used. The status of virus-infection of these plants with typical symptoms was verified by PCR detection, and the primers used here are listed in Additional file 1: Table S1.

**Fig. 1:**
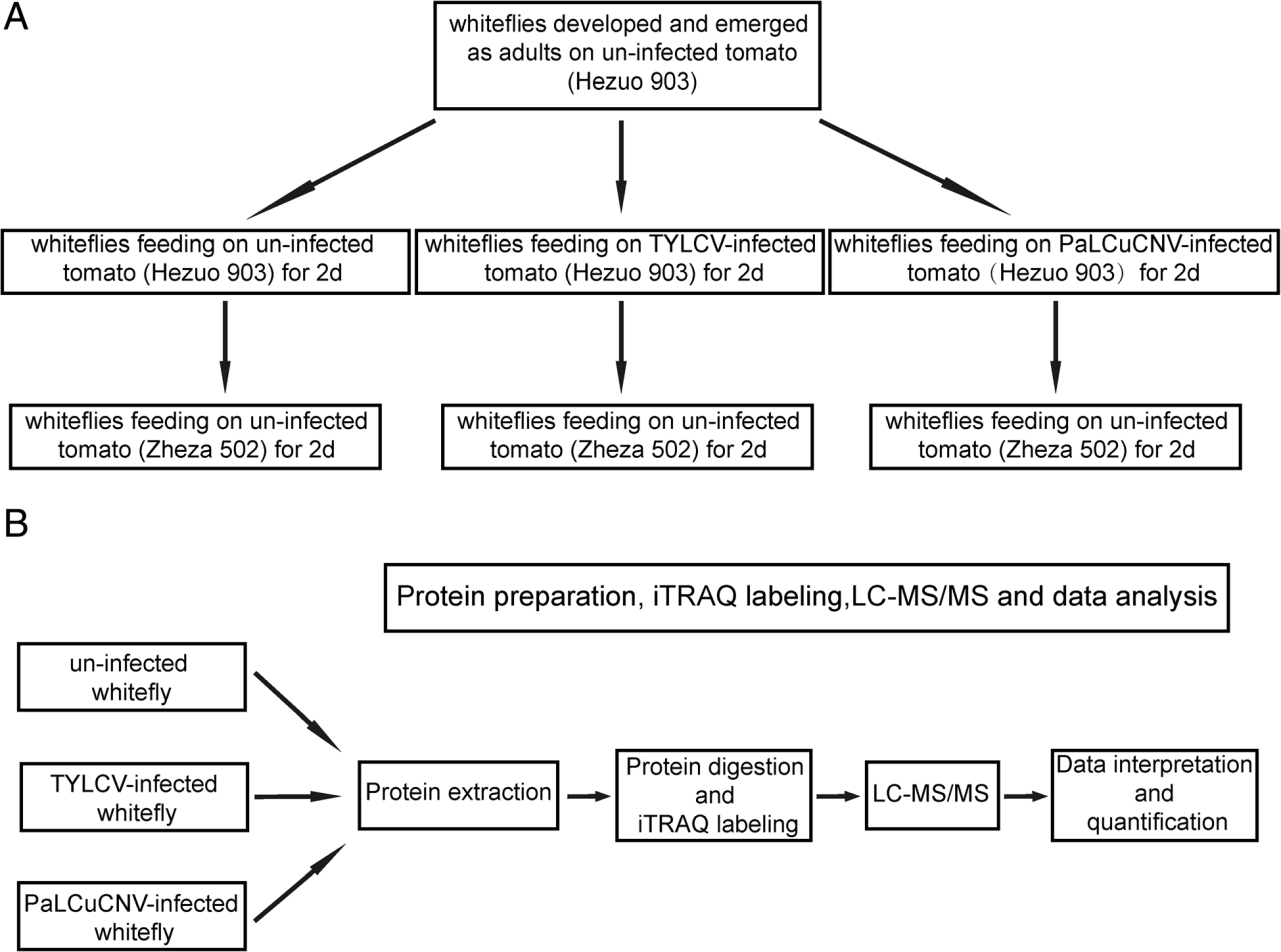
Workflow illustration. (**a**) Workflow for obtaining whiteflies used in the treatments. Two cultivars of tomato were used in this study, i.e. *S. lycopersicum* cv. Hezuo 903 (susceptible) and cv. Zheza 502 (resistant). For each of the three treatments, two biological replicates were conducted. All arrows indicate whitefly transfer. (**b**) Workflow for iTRAQ analysis.

To obtain whiteflies feeding on un-infected, TYLCV-infected and PaLCuCNV-infected plants, whitefly adults from the MED culture were collected 5–7 d post emergence, and then were placed on un-infected, TYLCV-infected and PaLCuCNV-infected tomato plants of Hezuo 903 respectively to feed for 2 d. The whitefly adults of each of the three treatments were then transferred to feed on un-infected tomato plants of a begomovirus-resistant cultivar (*S. lycopersicum* cv. Zheza 502) [35] for a further 2 d to reduce/eliminate effects of host plant differences on whiteflies during the 2d treatments (Fig. 1a). The virus-infection status of viruliferous whiteflies or no-viruliferous whiteflies was verified using PCR detection, and the primers used are listed in Additional file 1: Table S1. At this time, adults were collected for proteomics analysis (Fig. 1b). For each of the three treatments, two biological replicates in two separate cages were conducted. All whitefly cohorts of the three treatments were reared in cages at 25–27 °C, 60 ± 10% relative humidity and 14 h light/10 h darkness.

### Quantitative iTRAQ-LC-MS/MS proteomics analysis

For iTRAQ analysis, whitefly adults of the three treatments as described above, i.e. whiteflies feeding on un-infected, TYLCV-infected and PaLCuCNV-infected plants, were arranged into three combinations for comparison: (i) un-infected vs. TYLCV-infected, (ii) un-infected vs. PaLCuCNV-infected, and (iii) TYLCV-infected vs. PaLCuCNV-infected. For each of the two treatments in a combination for comparison, 0 .1g sample was taken for protein extraction. The methods and procedures of quantitative proteomics analyses followed those of Zhong et al [33]. Briefly, (i) protein extraction, digestion and iTRAQ labeling, (ii) LC-MS/MS analysis, and (iii) proteomic data analysis (Fig. 1b). The raw MS/MS data was converted into MGF format using ProteoWizard tool msConvert (version 3.0.1), and then peptides were identified by searching the MED transcriptomes. We used a MS/MS data interpretation algorithm within Mascot (version 2.3.02). At least one unique peptide was necessary for an identified protein. Based on the data of protein extraction, the bands and repeatability were qualified, and the total content of protein in each of the treatments was greater than 400 μg.

Differential expression ratios of proteins were analyzed by the automated software IQuant (version 2.2.1). To calculate differential expression ratios, all identified spectra from a protein were used to obtain an average protein ratio relative to the control label (i.e. fold change). Student t-test was used to analyze the differential expressed proteins between two treatments. We used *P <* 0.05 and the fold change > 1.2-fold or < 0.83 fold as the thresholds to judge the significance of differential expressed proteins. We used coefficient of variation (CV), which is defined as the ratio of the standard deviation (SD) to the mean, to evaluate reproducibility.

### Gene ontology, pathway enrichment, cluster analysis and cuticle protein family analysis

The identified proteins were categorized according to their Gene Ontology (GO) annotation (http://www.geneontology.org/). The metabolic pathway analysis of the proteins was conducted according to the Kyoto Encyclopedia of Genes and Genomes (KEGG) Pathway Database (http://www.genome.jp/kegg). The cluster analysis was conducted using the software Genesis (version 1.8.1). The cuticle protein family analysis was conducted using CutProtFam (aias.biol.uoa.gr/CutProtFam-Pred/home.php) [36].

### RT-qPCR validation

To validate results from iTRAQ analysis, genes encoding DEPs among the three treatments were subjected to the RT-qPCR analysis. Twenty adult whiteflies were collected as a group for analyzing the gene expression of DEPs, and three replicates were set for each treatment. For gene expression analysis, total RNA of whitefly was isolated by TRIzol (Ambion, USA) and reverse transcribed using PrimeScript RT reagent Kit (TaKaRa, Japan) following the manufacturer’s protocol. Quantitative PCR (qPCR) was performed on a CFX96™ Real-Time PCR Detection System (Bio-Rad, USA) with SYBR Premix ExTaq II (Takara, Japan). β-actin was used as internal reference, relative abundance of begomovirus or transcripts was calculated by 2^-ΔCt^. Primers used for real-time PCR are listed in Additional file 1: Table S1.

## Results

### Basic quantitative parameters

Among the 296,454 spectra generated, 46,699 spectra were identified with 42,153 being unique, 13,042 peptides were identified with 11,954 being unique as judged using 1% PSM (Peptide-spectrum matches) FDR (false discovery rate) (spectrum level), and 3555 proteins were identified with the 1% protein FDR protein levels. In the iTRAQ data, the values of CV exhibit centralized distributions within 0–10% (Additional file 2: Figure S1), indicating a fine reproducibility.

### Differentially expressed proteins (DEPs)

**Fig. 2:**
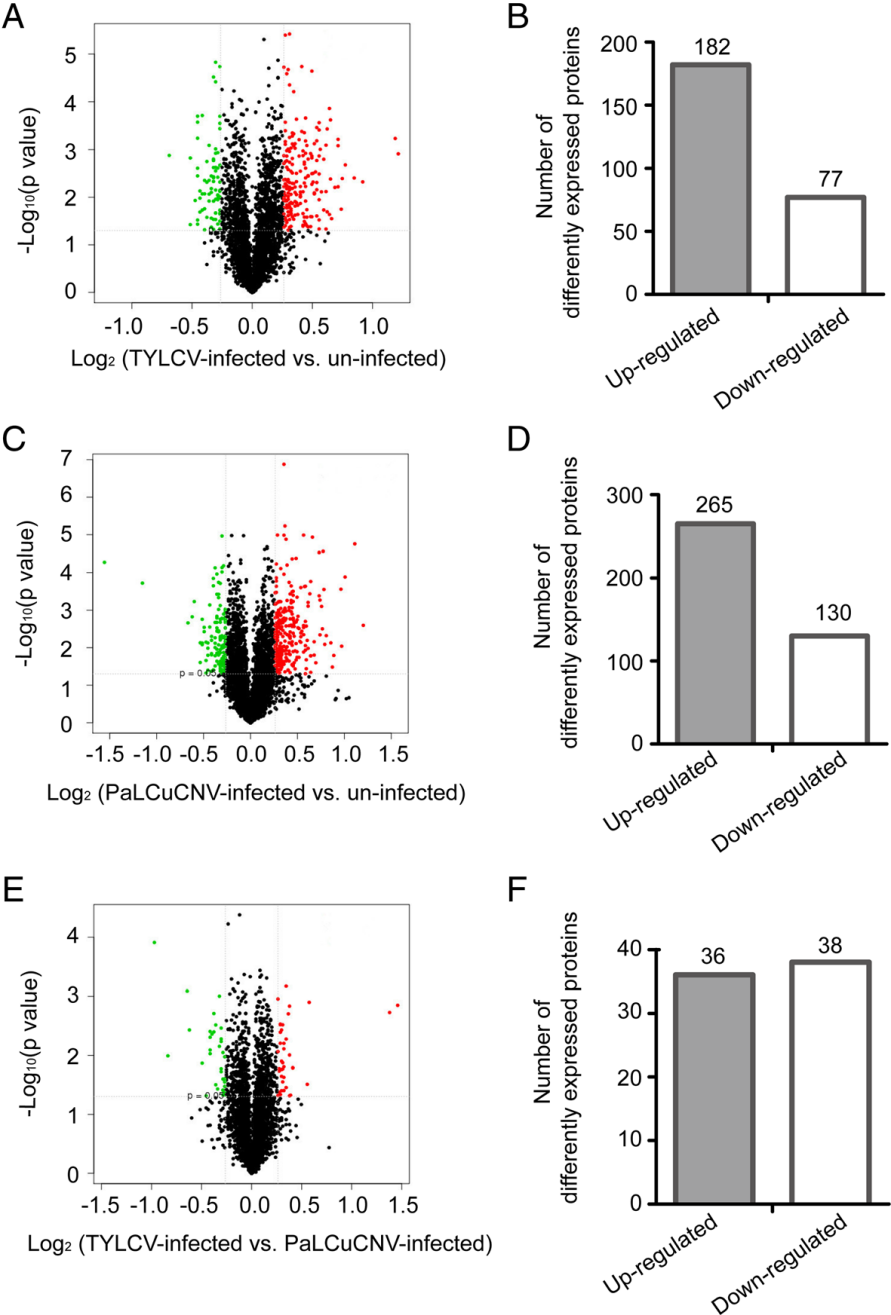
Protein expression patterns of MED whiteflies in response to viral infections. Protein expression patterns in whiteflies of TYLCV-infected vs. un-infected (**a**, **b**), PaLCuCNV-infected vs. un-infected (**c**, **d**), and TYLCV-infected vs. PaLCuCNV-infected (**e**, **f**). The Volcano figures of DEPs (**a**, **c** and **e**) depict volcano plot of log_2_ fold-change (x-axis) versus -log_10_ Q value (y-axis, representing the probability that the protein is differentially expressed) in each of the three combination for comparison. *P <* 0.05 and fold change *>* 1.2 were set as the significant threshold for differential expression. In each of the three diagrams of (**a**, **c**, and **e)**, the red dots indicate significantly up-regulations, and the green dots indicate significant down-regulations, while the black dots indicate no significant changes in regulations

**Fig. 3:**
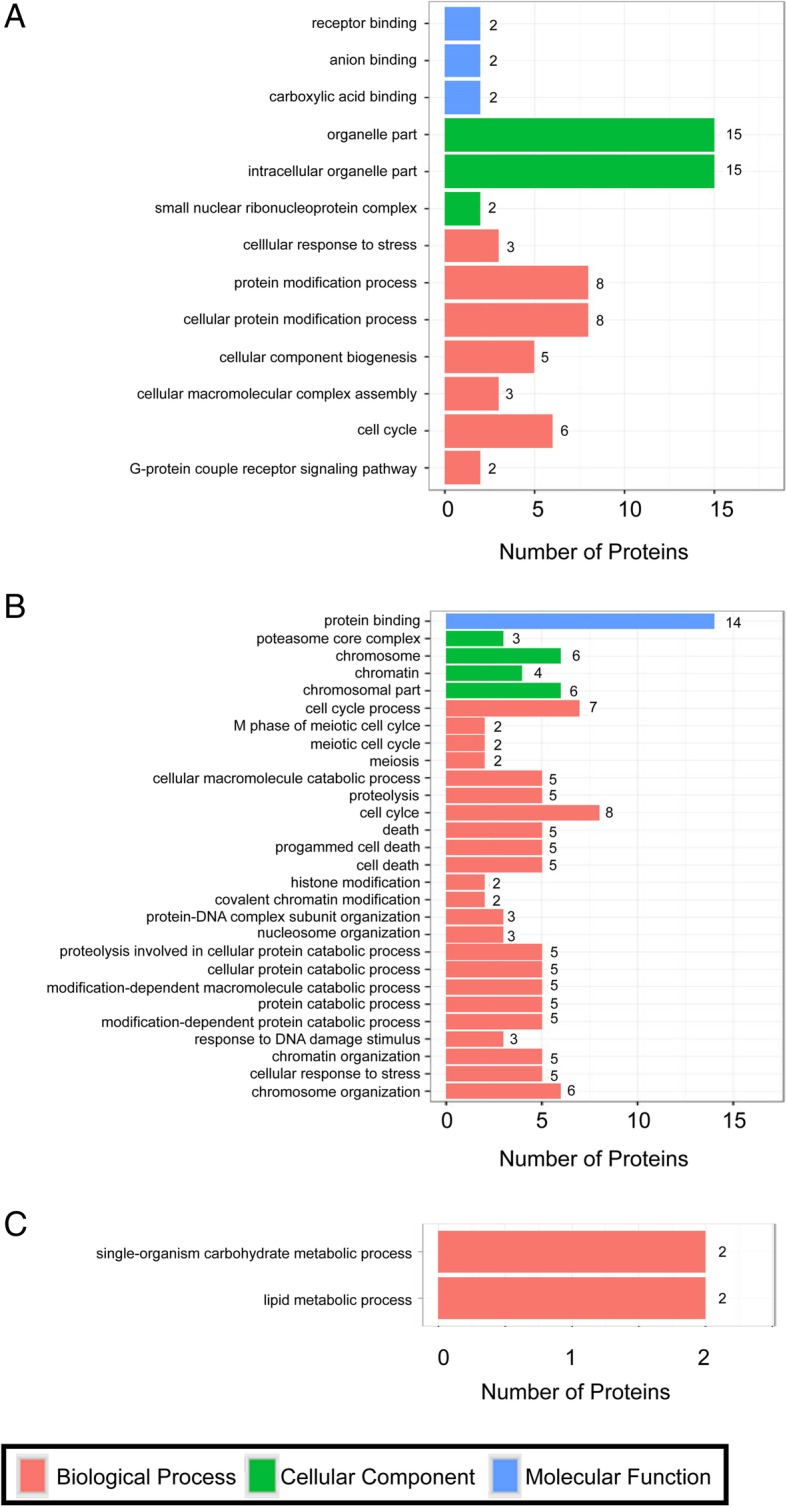
Gene ontology analysis. The bar chart shows the distribution of corresponding GO terms (*P <* 0.05). Different colors represent different GO categories. **a** TYLCV-infected vs. un-infected. **b** PaLCuCNV-infected vs. un-infected. **c** TYLCV-infected vs. PaLCuCNV-infected

**Fig. 4:**
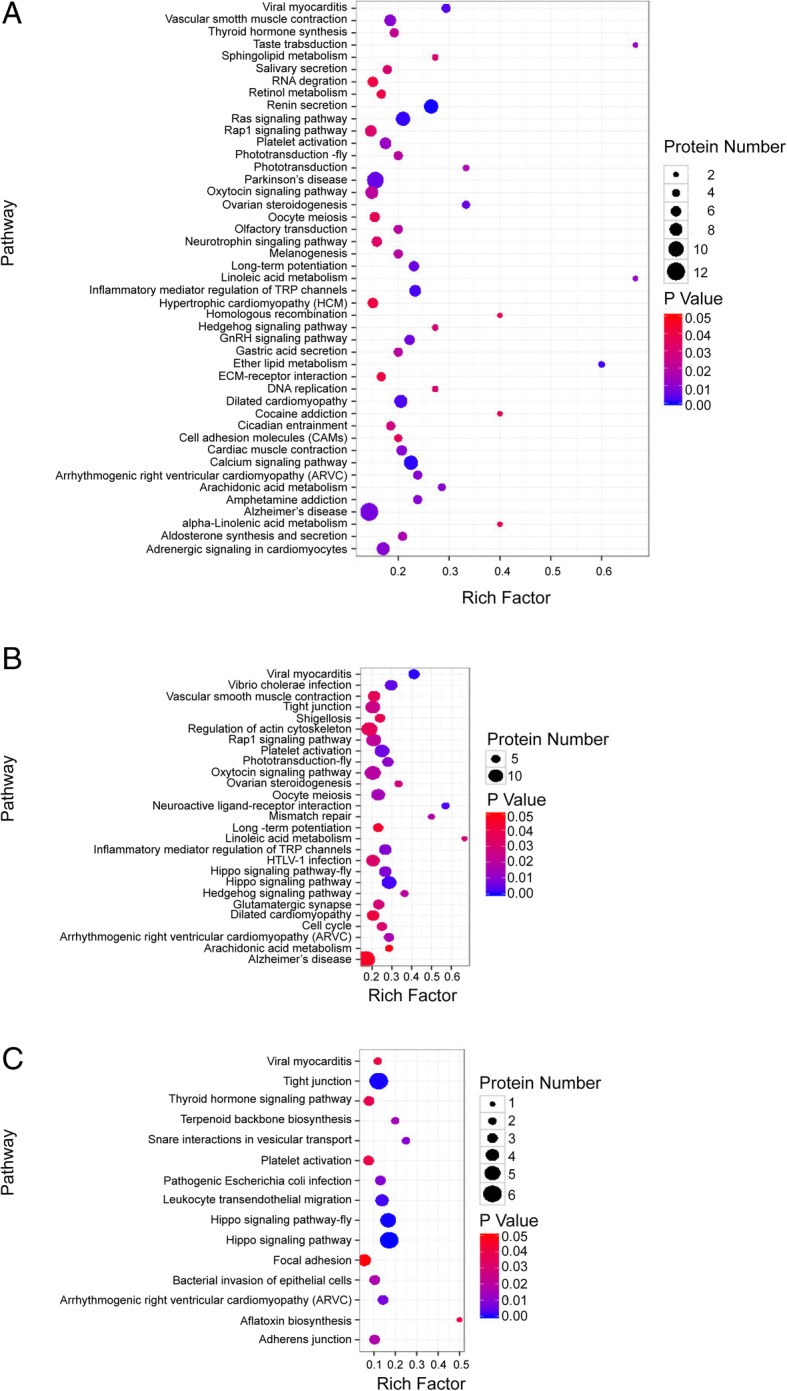
Pathway enrichment analysis. Rich factor is the ratio of differentially expressed protein number annotated in this pathway term to all protein number annotated. Greater rich factor means greater intensiveness. *P* value ranges from 0~1, and lower *P* value means greater intensiveness. Displayed here are enriched pathway terms with *P <* 0.05. **a** TYLCV-infected vs. un-infected, **b** PaLCuCNV-infected vs. un-infected, and **c** TYLCV-infected vs. PaLCuCNV-infected

For TYLCV-infected vs. un-infected whiteflies, 259 DEPs were identified with 182 being up-regulated and 77 down-regulated (Fig. 2a and b; Additional file 1: Table S2). For PaLCuCNV-infected vs. un-infected, 395 DEPs were identified with 265 being up-regulated and 130 down-regulated (Fig. 2c and d; Additional file 1: Table S3). For TYLCV-infected vs. PaLCuCNV-infected, 74 DEPs were identified with 36 being up-regulated and 38 down-regulated (Fig. 2e and f; Additional file 1: Table S4). Among the 259 DEPs in TYLCV-infected vs. un-infected, 93 were subcategorized into GO classes. These DEPs were found related to receptor group, protein modification process and other processes. Among the 395 DEPs evaluated in PaLCuCNV-infected vs. un-infected, 155 DEPs were subcategorized into GO classes. Most of them were involved in cell cycle, immune response and catabolic process. Among the 74 DEPs evaluated in TYLCV-infected vs. PaLCuCNV-infected, 29 DEPs were subcategorized into GO classes. Groups related to metabolic process exhibited significant enrichment. GO classes with *P* < 0.05 in each comparison are shown in Fig. 3. In addition, DEPs in the three comparisons were assigned to the reference pathways in KEGG. As a result, 189, 242 and 99 DEPs in the comparison of TYLCV-infected vs. un-infected, PaLCuCNV vs. un-infected, and TYLCV-infected vs. PaLCuCNV-infected were assigned to the reference pathways in KEGG respectively. Finally, 45, 27, 15 pathways were significantly enriched for whiteflies of TYLCV-infected vs. un-infected, PaLCuCNV-infected vs. un-infected, and TYLCV-infected vs. PaLCuCNV-infected, respectively (*P* < 0.05) (Fig. 4).

### Cluster analysis

**Fig. 5:**
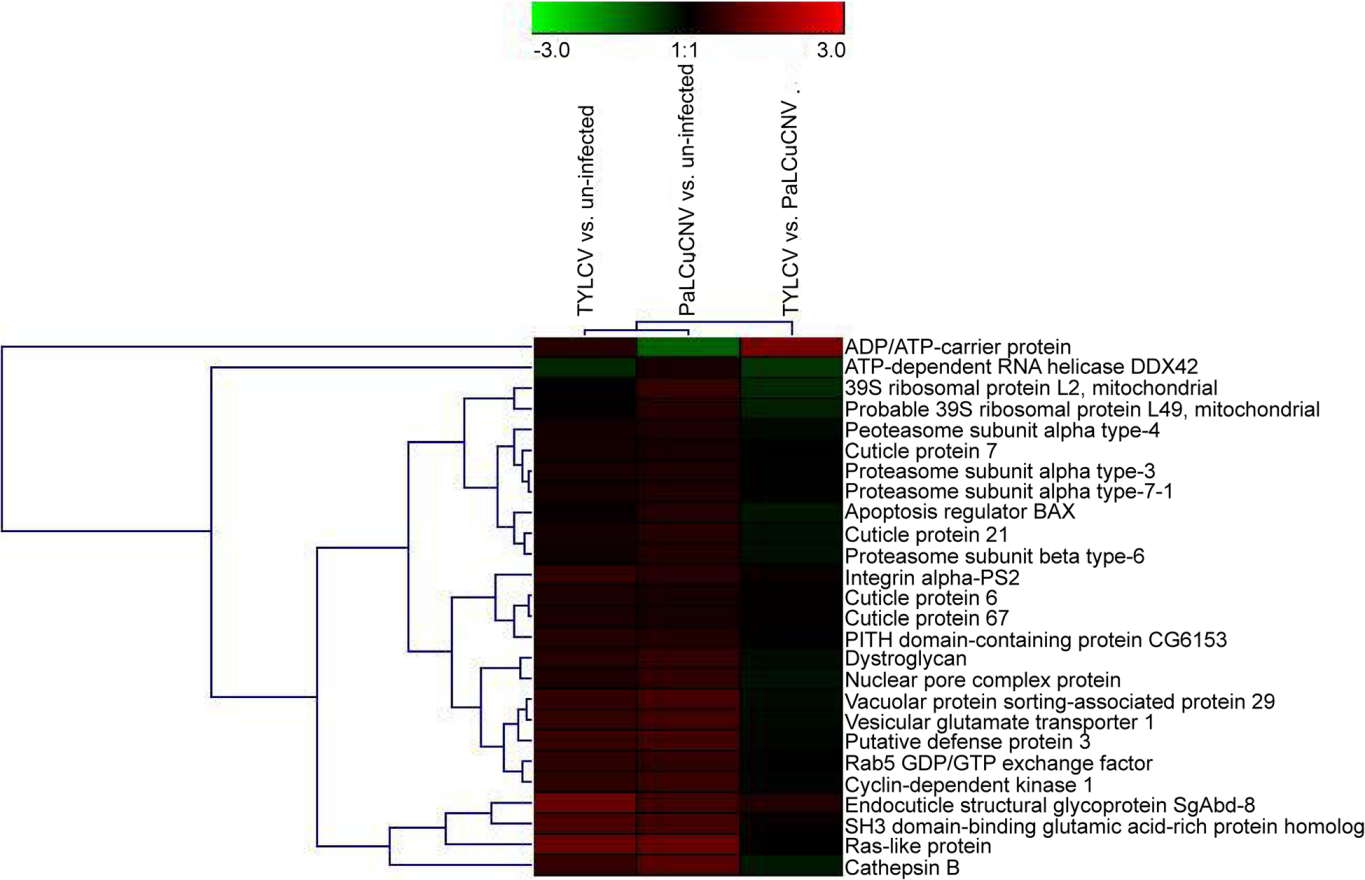
Hierarchical cluster analysis of DEPs. Fold changes of protein abundance in three combinations of comparison were analyzed using the software Genesis (version 1.8.1)

A cluster analysis of DEPs was conducted for the three comparisons (Fig. 5), including DEPs related to viral transport, defense response, cell cycle and other processes. Whiteflies infected with different begomoviruses showed some similar responses, including the pathway of ECM-receptor interactions (Table 1), and the up-regulations of some cuticle proteins (Table 2) and proteins related to defense responses (Table 3). In addition, some responses, such as 20S proteasome subunits, were up-regulated only in the comparison of PaLCuCNV-infected vs. un-infected whiteflies (Table 4).

**Table 1:** DEPs involved in the pathway of ECM-receptor interactions

Protein ID	Protein name	Fold change
TYLCV-infected vs. un-infected		
comp72848_c1_orf1	Dystroglycan	1.35
comp250437_c0_orf1	Integrin alpha-PS2	1.45
comp66903_c0_orf1	Integrin alpha-PS2-like	1.29
comp380948_c0_orf1	Laminin subunit alpha	1.36
comp38150_c0_orf1	Basement membrane proteoglycan	1.26
PaLCuCNV-infected vs. uninfected		
comp72848_c1_orf1	Dystroglycan	1.5
comp250437_c0_orf1	Integrin alpha-PS2	1.36
comp380948_c0_orf1	Laminin subunit alpha	1.41

**Table 2:** Up-regulated cuticle proteins in viruliferous whiteflies

Protein ID	Protein name	Fold change
TYLCV-infected vs. un-infected		
comp59667_c0_orf1	Cuticle protein 6	1.26
comp57333_c0_orf1	Cuticle protein 67, isoform A	1.25
comp67346_c0_orf1	Cuticle structural protein PCP16.7	1.22
PaLCuCNV-infected vs. un-infected		
comp61375_c0_orf1	Cuticle protein 21	1.35
comp45791_c0_orf1	Cuticle protein 7	1.21

**Table 3:** Up-regulated proteins related to defense responses in viruliferous whiteflies

Protein ID	Protein name	Fold change
TYLCV-infected vs. un-infected		
c63230_g1	Putative defense protein 3	1.57
comp74180_c0_orf1	PITH domain-containing protein CG6153	1.32
c64808_g1	Heat shock factor-binding protein 1	1.28
comp70278_c0_orf1	Multidrug resistance-associated protein 1	1.25
PaLCuCNV-infected vs. un-infected		
c63230_g1	Putative defense protein 3	1.66
comp74180_c0_orf1	PITH domain-containing protein CG6153	1.33
comp65735_c0_orf1	Apoptosis regulator BAX	1.31

**Table 4:** Up-regulated 20S proteasome subunits in the comparison of PaLCuCNV-infected vs. un-infected

Protein ID	Protein name	Fold change
comp59031_c0_orf1	Proteasome subunit beta type-6	1.31
comp75481_c0_orf1	Proteasome subunit alpha type-7-1	1.27
c44482_g1	Proteasome subunit alpha type-3	1.25
comp75391_c0_orf1	Proteasome subunit alpha type-4	1.24

### RT-qPCR validation of DEPs identified by proteomics

**Fig. 6:**
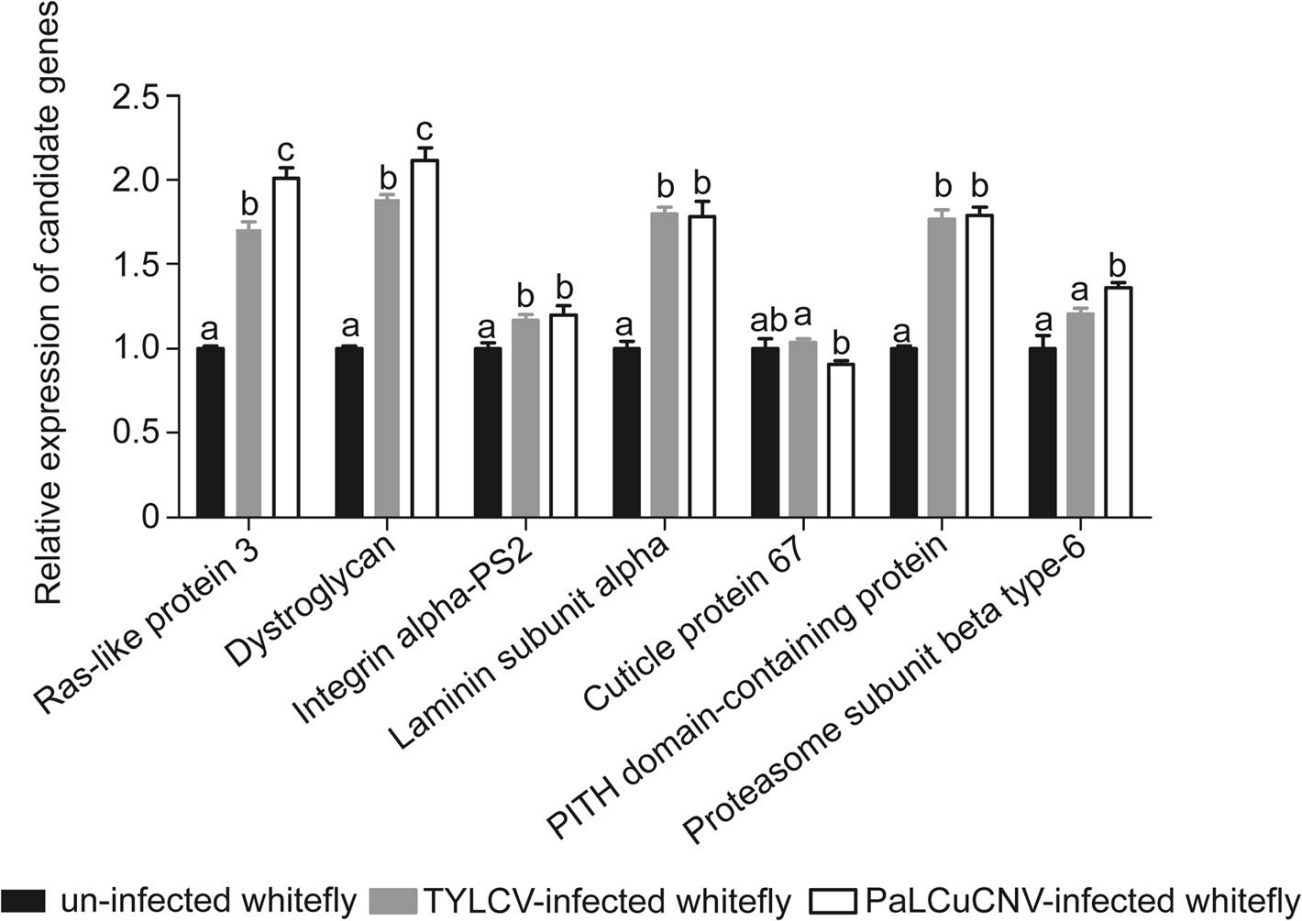
qPCR analysis of candidate genes. Values of control group (un-infected whitefly) were all set to 1.0 unit. Error bars represent the standard deviation. Significance is indicated with different letters; Student’s t-test

To validate the iTRAQ data, we tested the expression levels of some selected candidate genes in the three treatments. Figure 6 shows the expression patterns of 7 genes, including ras-like protein 3, dystroglycan, integrin alpha-PS2, laminin subunit alpha, cuticle protein 67, PITH domain-containing protein CG6153, and proteasome subunit beta type-6. Consistent with the iTRAQ data, ras-like protein 3, dystroglycan, integrin alpha-PS2, laminin subunit alpha and PITH domain-containing protein were significantly up-regulated after TYLCV/PaLCuCNV infection compared with un-infected whiteflies. Proteasome subunit beta type-6, a kind of 20S proteasome, was significantly up-regulated in the comparison of PaLCuCNV vs. un-infected whiteflies, a result in agreement with the iTRAQ data. However, no significant change was observed in the mRNA expression level of cuticle protein 67.

## Discussion

### Three cellular viral receptors induced by TYLCV and PaLCuCNV infections

Binding of a virus to cellular receptors is the key determinant of the physiological outcome of infection [37]. To infect vector cells, viruses need to gain access to the cell surface and then bind their receptor (s). Some attachment factors are required for allowing viral concentration at the cell surface, and following this primary attachment, the viral interaction with specific receptors permits its internalization. This process often requires more than one receptor [38, 39]. A previous study demonstrated that clathrin-mediated endocytosis was involved in TYLCV transport across the vector midgut wall [10], but the receptors that mediated this process remain unknown. According to our iTRAQ data, the expressions of laminin subunit alpha, dystroglycan and integrin alpha-PS2, three proteins in the pathway of extracellular matrix (ECM)-receptor interactions, were significantly up-regulated by 1.36, 1.35 and 1.45 fold respectively in the comparison of TYLCV-infected vs. un-infected whiteflies. Similarly, in the comparison of PaLCuCNV-infected vs. un-infected whiteflies, these three proteins were significantly up-regulated by 1.41, 1.5, 1.36 fold respectively. Laminin has been stated as a cellular attachment receptor for some animal viruses [40, 41]. Alpha-dystroglycan is a type of important cellular viral receptor [37, 42–44]. Many integrin subunits have been reported to be usurped by a number of viral and bacterial pathogens in order to gain entrance into host cells [45–48]. Then, laminin subunit alpha, dystroglycan and integrin alpha-PS2, and three enriched cell surface receptors identified through our iTRAQ analysis after begomovirus infection, may synergistically regulate the endocytosis of begomovirus infection. The fact that clathrin-mediated endocytosis can affect virus penetration of the vector midgut epidermal cells implies that the receptor-mediated endocytosis was essential for viral transmission.

### Cuticle proteins induced by TYLCV and PaLCuCNV infection

According to the proteins of insects that have a complete genome, over 1% of the total proteins are cuticle proteins [49], indicating the importance of cuticle proteins in insect body. In our iTRAQ data, three cuticle proteins, cuticle protein 6 (1.26-fold), cuticle protein 67, isoform A (1.25-fold) and cuticle structural protein PCP16.7 (1.22-fold), were significantly up-regulated in the comparison of TYLCV-infected vs. un-infected whiteflies, and two cuticle proteins, cuticle protein 21 (1.35-fold) and cuticle protein 7 (1.21-fold), were significantly up-regulated in the comparison of PaLCuCNV-infected vs. un-infected whiteflies. No cuticle proteins showed down-regulation in both comparisons. According to the analysis from CutProtFam, cuticle protein 6 and cuticle protein 21 belong to the CPR family and RR-2 subgroup, cuticle protein 67, isoform A belongs to CPF family. However, none of the cuticle protein families or sub-families was identified when referring to cuticle structural protein PCP16.7 and cuticle protein 7. For a persistently transmitted virus, e.g. *cereal yellow dwarf virus*-*Rhopalosiphum padi virus*, at least four cuticular proteins are involved in the transmission process by the greenbug aphid, *Schizaphis graminum* [50]. In addition, *rice stripe virus* can utilize a hemipteran cuticular protein of the small planthopper, *Laodelphax striatellus*, to facilitate its survival in the hemolymph [51]. So far, the functions of cuticle proteins in begomoviruses transmission have not been reported. Here, we provide a reference for further studies on, for example, clarification of functions of these cuticle proteins identified from our data in begomovirus transmission.

### Similar defense responses induced by TYLCV and PaLCuCNV infection

Although insects lack an adaptive immune system, they possess internal defense mechanisms when facing foreign pathogens [52, 53]. In whiteflies, several components have been reported to play a role in viral response, such as the heat shock protein 70 protein and autophagy pathway [14, 54]. According to our iTRAQ data coupled with GO and pathway analysis, in the comparison of TYLCV-infected vs. un-infected whiteflies, some proteins related to defense response, including putative defense protein 3, PITH domain-containing protein CG6153, heat shock factor-binding protein 1 and multidrug resistance-associated protein 1, were significantly up-regulated by 1.57, 1.32, 1.28, 1.25 fold respectively. Similarly, in the comparison of PaLCuCNV-infected vs. un-infected whiteflies, putative defense protein 3, PITH domain-containing protein CG6153 and apoptosis regulator BAX were significantly up-regulated by 1.66, 1.33, 1.31 fold respectively.

### Different defense responses induced by TYLCV and PaLCuCNV infection

A given whitefly species can often acquire and transmit different begomoviruses with varied efficiencies [9]. The different characteristics of TYLCV and PaLCuCNV transmission by MED indicate: (i) following viral acquisition, TYLCV can accumulate in the whitefly but PaLCuCNV is unable to [54], and (ii) PaLCuCNV penetrates through the midgut wall of MED less efficiently than TYLCV, resulting in a lower efficiency of PaLCuCNV transmission by MED [11, 24]. In our iTRAQ data, four 20S proteasome subunits were significantly up-regulated in the comparison of PaLCuCNV-infected vs. un-infected whiteflies, namely proteasome subunit beta type-6 (1.31-fold), proteasome subunit alpha type-7-1 (1.27-fold), proteasome subunit alpha type-3 (1.25-fold), and proteasome subunit alpha type-4 (1.24-fold). Interestingly, no 20S or related proteasome subunits showed significant changes in the comparison of TYLCV-infected vs. un-infected whiteflies. Host proteasome-mediated protein proteolysis is known as a common strategy used by both plants and animals for virus degradation and accumulation [55–57]. The proteasomes are large multi-subunit proteinase complexes and exist as particles of 20S and of 26S. The 20S particle of ≈700 kDa is an important component of 26S complex of ≈2000 kDa, which is responsible for the degradation of many cellular proteins as a proteolytic core [58, 59]. Ubiquitin-proteasome system has been reported to limit the quantity of begomovirus in whitefly [60]. Thus, in consideration of the potential antiviral function of 20S proteasome, the up-regulated 20S proteasome subunits in PaLCuCNV-infected vs. un-infected whiteflies may be one important factor that leads to the failure of PaLCuCNV accumulation in whitefly body and in turn the lower level of PaLCuCNV acquisition and transmission by MED whitefly.

## Conclusions

Previous studies on the interactions between begomoviruses and whiteflies indicate that PaLCuCNV penetrates through the midgut wall of MED whitefly less efficiently than that of Middle East-Asia Minor 1 (MEAM1) whitefly, resulting in a lower efficiency of PaLCuCNV acquisition and transmission by MED than that by MEAM1 [11]. In view of the circulative journey of begomovirus in whitefly [61], both the poorer ability of PaLCuCNV to bind to the midgut cells in MED whitefly and the more sensitive defense responses in MED whitefly could lead to this difference. The data in this study suggests that MED whiteflies infected by TYLCV and PaLCuCNV share some membrane and transport proteins as well as some defense proteins. However, the changes of 20S proteasome subunits between the comparison of TYLCV-infected vs. un-infected whiteflies and PaLCuCNV-infected vs. un-infected whiteflies were in a completely different way. In the future, dsRNA interference could be used to test the roles of laminin subunit alpha, dystroglycan, integrin alpha-PS2 and cuticle proteins in transmission of TYLCV and PaLCuCNV. Taken together, our findings provide new insight into the interactions between whiteflies and begomoviruses, which will serve to provide a number of putative proteins for future investigation on mechanisms underlying whitefly transmission of begomoviruses at the proteome level.

## Additional files

Additional file 1: **Table S1.** Primers used in this study. **Table S2.** DEPs identified in the comparison of TYLCV-infected vs. un-infected. **Table S3**. DEPs identified in the comparison of PaLCuCNV-infected vs. un-infected. **Table S4.** DEPs identified in the comparison of TYLCV-infected vs. PaLCuCNV-infected. (DOCX 61 kb)

Additional file 2: **Figure S1.** CV distribution of replicates in each of the three combinations for comparison. X-axis is the deviation between the protein ratio of the repeated samples. Y-axis is the percentage of proteins at a certain angle with given levels of quantified proteins. CV value = SD/mean, the lower the value, the better the replication. (A) CV distributions in the comparison of TYLCV-infected vs. un-infected, (B) CV distributions in the comparison of PaLCuCNV-infected vs. un-infected, (C) CV distributions in the comparison of TYLCV-infected vs. PaLCuCNV-infected. (PDF 199 kb)

## Abbreviations

CV: Coefficient of variation
DEPs: Differentially expressed proteins
FDR: False discovery rate
GO: Gene Ontology
iTRAQ: Isobaric tags for relative and absolute quantification
KEGG: Kyoto Encyclopedia of Genes and Genomes
LC-MS/MS: Liquid chromatography-tandem mass spectrometry
MED: Mediterranean
PaLCuCNV: Papaya leaf curl China virus
PSM: Peptide-spectrum matches
SD: Standard deviation
TYLCV: Tomato yellow leaf curl virus
