# Supplementary material for: Comparative proteomic analysis provides new insight into differential transmission of two begomoviruses by a whitefly
Source: Virol J. 2019 Mar 11;16:32. doi: 10.1186/s12985-019-1138-4 (PMC6413443; doi:10.1186/s12985-019-1138-4)
Supplement: Supplementary file 1 — Table S1. Primers used in this study. Table S2. DEPs identified in the comparison of TYLCV-infected vs. un-infected. Table S3. DEPs identified in the comparison of PaLCuCNV-infected vs. un-infected. Table S4. DEPs identified in the comparison of TYLCV-infected vs. PaLCuCNV-infected. (DOCX 61 kb) [file 12985_2019_1138_MOESM1_ESM.docx]

**Additional file 1**

**Comparative proteomic analysis provides new insight into differential transmission of two begomoviruses by a whitefly**

Jing Zhao^*^, Yao Chi^*^, Xin-jia Zhang, Teng Lei, Xiao-wei Wang, Shu-sheng Liu

^*^ Equal contribution

Ministry of Agriculture Key Laboratory of Molecular Biology of Crop Pathogens and Insects, Institute of Insect Sciences, Zhejiang University, Hangzhou 310058, P.R. China

**Table S1. Primers used in this study.**

| **Primer Name** | **Sequence** |
| --- | --- |
| TYLCV-detection-F | ATCGAAGCCCTGATGTTCCTCGTGG |
| TYLCV-detection-R | CAGAGCAGTTGATCATGTATTGTATG |
| PaLCuCNV-detection-F | TAGTCATTTCCACTCCCGC |
| PaLCuCNV-detection-R | TGATTGTCATACTTCGCAGC |
| ras-like-qPCR-F | GTTCCGTTCCAGCAGTGTCTA |
| ras-like-qPCR-R | TTGCATAGTTCGATGTTGTCGCA |
| Dystroglycan-qPCR-F | TGCAACCGTTGAGTCAACAC |
| Dystroglycan-qPCR-R | CAGGATCAAAAGTTGGTGTT |
| integrinα-PS2-q-F | GGTCTTGGCGCCTCCATCTC |
| integrinα-PS2-q-R | GCTGGTGGCCCTTCATTCGT |
| Laminin-qPCR-F | AGGCCAGTGCAAGTGCAAAC |
| Laminin-qPCR-R | AGGGTTGCAGCCTACACCAA |
| Cuticle67-qPCR-F | CCCGCAGGTTTACTACTCG |
| Cuticle67-qPCR-R | CGGAGGATGTTGGAGGACT |
| PITH-qPCR-F | TGCGGTTGAGTGTCTCAATG |
| PITH-qPCR-R | CTGCGTCAGCATCACTTTCA |
| proteasome 6-qPCR-F | AGGAGTACAAAGAAGGCATG |
| proteasome 6-qPCR-R | TGATGATACCGATTCTTG |

**Table S2. DEPs identified in the comparison of TYLCV-infected vs. un-infected.**

| Protein ID | Protein name | Fold change |
| --- | --- | --- |
| **Up-regulated** |  |  |
| comp73512_c0_orf1 | hypothetical protein SINV_15050 | 2.32 |
| comp67637_c0_orf1 | PREDICTED: ras-related protein Rap-1b-like isoform 1 | 2.28 |
| c67729_g1 | PREDICTED: similar to phospholipase A2, group VI (cytosolic, calcium-independent) | 1.89 |
| c38279_g1 | PREDICTED: SH3 domain-binding glutamic acid-rich protein homolog | 1.8 |
| comp73324_c0_orf1 | PREDICTED: heterogeneous nuclear ribonucleoprotein K-like isoform 1 | 1.71 |
| comp58988_c0_orf1 | AAEL010801-PB | 1.68 |
| comp65705_c0_orf1 | PREDICTED: retinol dehydrogenase 11-like isoform 1 | 1.67 |
| comp74417_c0_orf1 | hypothetical protein TcasGA2_TC004221 | 1.64 |
| comp66486_c0_orf1 | PREDICTED: elongation of very long chain fatty acids protein 4-like | 1.64 |
| c64110_g1 | splicing factor, arginine/serine-rich 2 | 1.64 |
| c63913_g1 | hypothetical protein KGM_22666 | 1.61 |
| comp73067_c1_orf1 | farnesoic acid methyltransferase | 1.58 |
| c63230_g1 | putative defense protein 3 | 1.57 |
| c61923_g1 | nuclear protein | 1.57 |
| c68324_g2 | unknown | 1.56 |
| c153250_g1 | GH22984 | 1.56 |
| comp60292_c0_orf1 | TPA_inf: cathepsin B | 1.54 |
| comp61888_c0_orf1 | sphingosine kinase A, B, putative | 1.54 |
| c66096_g5 | conserved hypothetical protein | 1.53 |
| c59925_g1 | 39S ribosomal protein L53, mitochondrial | 1.53 |
| c51657_g1 | AGAP007992-PA | 1.53 |
| c63170_g1 | PREDICTED: vacuolar protein sorting-associated protein 29-like | 1.51 |
| comp67402_c3_orf2 | D-beta-hydroxybutyrate dehydrogenase, putative | 1.51 |
| c69034_g2 | PREDICTED: similar to AGAP006340-PC | 1.49 |
| comp41022_c0_orf1 | vitellogenin-1 | 1.48 |
| comp73950_c0 | - | 1.47 |
| c57073_g1 | unnamed protein product | 1.47 |
| comp63595_c0_orf1 | PREDICTED: hypothetical protein LOC100166785 | 1.47 |
| comp66975_c0 | - | 1.47 |
| comp58993_c0_orf1 | cdk1 | 1.47 |
| comp55754_c0_orf1 | conserved hypothetical protein | 1.47 |
| comp72309_c1_orf1 | PREDICTED: similar to rab gdp/gtp exchange factor isoform 1 | 1.47 |
| c61243_g1 | GI16849 | 1.45 |
| comp41074_c1_orf1 | PREDICTED: small ubiquitin-related modifier-like | 1.45 |
| comp250437_c0_orf1 | integrin alpha-PS2 precursor, putative | 1.45 |
| comp56573_c1_orf1 | PREDICTED: dephospho-CoA kinase domain-containing protein-like | 1.45 |
| c67538_g1 | methionine sulfoxide reductase B3 isoform 1 | 1.45 |
| comp76302_c0_orf1 | membrane-associated protein, putative | 1.45 |
| c20768_g1 | vitellogenin-1 | 1.44 |
| comp52386_c0_orf1 | PREDICTED: DNA polymerase delta subunit 2-like | 1.43 |
| c61021_g1 | RNA polymerase-associated protein LEO1, putative | 1.43 |
| c59283_g1 | PREDICTED: synaptosomal-associated protein 25-like | 1.43 |
| c64603_g2 | hypothetical protein DAPPUDRAFT_42659 | 1.42 |
| c30353_g1 | orcokinin | 1.42 |
| c65325_g2 | - | 1.41 |
| WP_014895133_1 | symbionin symS | 1.41 |
| c1950_g1 | putative ribosomal protein S25 | 1.41 |
| c57402_g1 | hypothetical protein KGM_07372 | 1.41 |
| c69805_g9 | ACYPI38240 | 1.41 |
| comp67813_c0_orf1 | PREDICTED: hypothetical protein LOC100166909 | 1.41 |
| 64195_g1 | PREDICTED: sodium/calcium exchanger 1-like | 1.4 |
| comp40938_c0_orf1 | putative mitochondrial NADH dehydrogenase (ubiquinone) 1 alpha subcomplex | 1.4 |
| c56033_g1 | PREDICTED: similar to metaxin 2 | 1.4 |
| comp71236_c0_orf1 | PREDICTED: surfeit locus protein 1-like | 1.38 |
| comp63077_c0_orf1 | AT15141p | 1.38 |
| comp74852_c0_orf1 | hypothetical protein G5I_03899 | 1.38 |
| comp69323_c1 | - | 1.38 |
| c59207_g2 | - | 1.37 |
| c70930_g4 | - | 1.37 |
| c25716_g1 | low-density lipoprotein receptor, putative | 1.37 |
| comp72185_c0_orf1 | PREDICTED: prostatic acid phosphatase-like | 1.37 |
| c45818_g1 | AGAP003184-PA | 1.36 |
| comp70776_c1_orf1 | PREDICTED: protein alan shepard-like | 1.36 |
| c3648_g1 | PREDICTED: similar to ATP synthase delta chain, mitochondrial | 1.36 |
| comp477134_c0_orf1 | PREDICTED: hemicentin-1-like isoform 2 | 1.36 |
| comp380948_c0_orf1 | PREDICTED: laminin subunit alpha-like | 1.36 |
| comp879424_c0_orf1 | PREDICTED: chaoptin-like | 1.36 |
| comp70218_c0_orf1 | PREDICTED: similar to tau CG31057-PA | 1.36 |
| c67104_g2 | PREDICTED: hypothetical protein LOC100114408 | 1.36 |
| c62328_g1 | hypothetical protein DAPPUDRAFT_329664 | 1.35 |
| comp72848_c1_orf1 | conserved hypothetical protein | 1.35 |
| c51220_g1 | PREDICTED: icarapin-like | 1.35 |
| c63324_g1 | conserved hypothetical protein | 1.35 |
| comp71497_c1_orf1 | PREDICTED: cAMP-dependent protein kinase catalytic subunit-like | 1.35 |
| c35339_g1 | - | 1.35 |
| comp39853_c0 | - | 1.34 |
| c62046_g1 | unnamed protein product | 1.34 |
| comp67502_c0_orf1 | PREDICTED: UDP-glucuronosyltransferase 1-7-like | 1.34 |
| comp68485_c0_orf1 | PREDICTED: short-chain dehydrogenase/reductase family 16C member 6-like isoform 1 | 1.34 |
| fig\|572265_7_peg_974 | - | 1.34 |
| c61942_g1 | - | 1.34 |
| c69852_g2 | sjoegren syndrome nuclear autoantigen 1-like protein | 1.33 |
| c68842_g2 | PREDICTED: similar to groucho protein | 1.33 |
| comp647510_c0_orf1 | hypothetical protein KGM_12959 | 1.32 |
| comp1063366_c0_orf1 | ADP/ATP-carrier protein, partial | 1.32 |
| c52720_g1 | 60S ribosomal protein L27a | 1.32 |
| comp59199_c0_orf1 | PREDICTED: similar to conserved hypothetical protein | 1.32 |
| c69790_g2 | PREDICTED: hypothetical protein LOC100121386 isoform 1 | 1.32 |
| comp74180_c0_orf1 | PREDICTED: similar to AGAP010237-PA | 1.32 |
| c57159_g1 | GJ20513 | 1.32 |
| comp63139_c0_orf1 | 60S ribosomal protein L8-like | 1.31 |
| c48270_g1 | ACYPI008219 | 1.31 |
| c16261_g1 | PREDICTED: similar to maggie CG14981-PA | 1.3 |
| comp40774_c0_orf1 | PREDICTED: polyribonucleotide nucleotidyltransferase-like | 1.3 |
| c56539_g1 | uncharacterized protein LOC100163911 | 1.29 |
| comp66903_c0_orf1 | PREDICTED: integrin alpha-PS2-like | 1.29 |
| c68868_g2 | - | 1.29 |
| c65422_g1 | - | 1.29 |
| comp67800_c0_orf1 | Thioredoxin domain-containing protein 12 precursor | 1.29 |
| c64808_g1 | heat shock factor binding protein 1-like | 1.28 |
| comp29968_c0_orf1 | PREDICTED: hypothetical protein LOC100746188 | 1.28 |
| c59011_g1 | death-associated protein-like protein | 1.28 |
| comp59163_c0_orf1 | conserved hypothetical protein | 1.28 |
| c123606_g1 | lsm4 protein | 1.28 |
| c70349_g1 | cysteine string protein | 1.28 |
| c12520_g1 | vascular cell adhesion protein 1 precursor, putative | 1.28 |
| comp70506_c0_orf1 | PREDICTED: caspase-2-like isoform 1 | 1.27 |
| c65822_g1 | - | 1.27 |
| c63201_g1 | PREDICTED: WD40 repeat-containing protein SMU1-like isoform 1 | 1.27 |
| comp40702_c0_orf1 | hypothetical protein DAPPUDRAFT_110732 | 1.27 |
| c44015_g1 | PREDICTED: tropomyosin-2-like isoform 2 | 1.27 |
| comp71783_c1_orf1 | putative chemosensory protein CSP6 | 1.26 |
| comp74364_c0_orf1 | AGAP006662-PA | 1.26 |
| c64336_g1 | nuclear pore complex protein nup154, putative | 1.26 |
| comp72119_c0_orf1 | stromal interaction molecule-like protein | 1.26 |
| comp65802_c0_orf1 | PREDICTED: similar to succinate semialdehyde dehydrogenase, mitochondrial | 1.26 |
| c59346_g1 | actin | 1.26 |
| c43129_g1 | DPY-30 protein, putative | 1.26 |
| comp38150_c0_orf1 | PREDICTED: basement membrane-specific heparan sulfate proteoglycan core protein-like | 1.26 |
| comp59667_c0_orf1 | PREDICTED: hypothetical protein LOC100159939 | 1.26 |
| c61447_g1 | membrane-bound trehalase | 1.26 |
| comp67785_c0_orf1 | replication protein A 70 kDa DNA-binding subunit | 1.25 |
| comp59349_c0_orf1 | PREDICTED: putative rRNA methyltransferase 3-like | 1.25 |
| comp56975_c0_orf1 | histone H2AV | 1.25 |
| comp70278_c0_orf1 | PREDICTED: multidrug resistance-associated protein 1-like isoform 3 | 1.25 |
| c68596_g1 | PREDICTED: hypothetical protein LOC100119750 | 1.25 |
| comp60414_c0 | - | 1.25 |
| c1880_g1 | PREDICTED: protein LSM12 homolog | 1.25 |
| comp57333_c0_orf1 | Pupal cuticle protein C1B, putative | 1.25 |
| c57836_g3 | PREDICTED: hypothetical protein LOC100744819 | 1.25 |
| comp56451_c0_orf1 | PREDICTED: 60S ribosomal protein L7a-like | 1.24 |
| comp74974_c0_orf1 | hypothetical protein DAPPUDRAFT_306461 | 1.24 |
| c54345_g1 | signal recognition particle | 1.24 |
| c4493_g1 | cytochrome b-c1 complex subunit 8 | 1.24 |
| c66677_g1 | PREDICTED: tRNA pseudouridine synthase-like 1-like | 1.24 |
| comp67133_c0_orf1 | PREDICTED: glucose dehydrogenase [acceptor]-like | 1.24 |
| comp61231_c0_orf1 | PREDICTED: hypothetical protein LOC100647906 | 1.24 |
| c72114_g1 | predicted protein | 1.24 |
| c58393_g1 | - | 1.24 |
| comp71822_c0_orf1 | EH domain-binding protein 1 | 1.24 |
| c65636_g1 | PREDICTED: hypothetical protein LOC100741880 | 1.24 |
| c69335_g1 | PREDICTED: neuroblastoma-amplified sequence-like isoform 1 | 1.24 |
| comp58670_c0_orf1 | hypothetical protein DAPPUDRAFT_100476 | 1.24 |
| comp64445_c0_orf1 | CG12163, isoform B | 1.23 |
| comp67571_c0_orf1 | lingerer, putative | 1.23 |
| comp71764_c0_orf1 | PREDICTED: thiamin pyrophosphokinase 1-like | 1.23 |
| c41383_g1 | alpha-I tubulin | 1.23 |
| c58616_g1 | PREDICTED: pleckstrin homology domain-containing family F member 2-like isoform 3 | 1.23 |
| c66686_g2 | - | 1.23 |
| comp59039_c0 | - | 1.23 |
| comp70509_c0_orf1 | PREDICTED: hypothetical protein LOC100167736 | 1.23 |
| c64495_g1 | calcyphosin-like protein | 1.23 |
| c68616_g1 | - | 1.23 |
| c55608_g1 | troponin C-like | 1.23 |
| c70264_g1 | GE10211 | 1.23 |
| comp64748_c0_orf1 | PREDICTED: similar to S14e ribosomal protein | 1.22 |
| comp69527_c0 | - | 1.22 |
| comp29955_c0_orf1 | ribosomal protein L28-like protein | 1.22 |
| comp74679_c0_orf1 | - | 1.22 |
| comp62891_c0_orf1 | hypothetical protein TcasGA2_TC013378 | 1.22 |
| comp69910_c0_orf1 | hypothetical protein TcasGA2_TC006332 | 1.22 |
| c70437_g2 | PREDICTED: protein lin-7 homolog B-like | 1.22 |
| comp67346_c0_orf1 | hypothetical protein DAPPUDRAFT_259342 | 1.22 |
| comp67677_c0_orf1 | phosphatidylethanolamine-binding protein | 1.22 |
| comp64164_c0_orf1 | lysine-specific histone demethylase, putative | 1.22 |
| c63669_g1 | - | 1.22 |
| c59927_g1 | PREDICTED: probable enoyl-CoA hydratase, mitochondrial-like | 1.22 |
| c121590_g1 | hypothetical protein TcasGA2_TC012992 | 1.22 |
| comp63573_c0_orf1 | putative ribosomal protein S23e | 1.21 |
| comp68746_c0_orf1 | hypothetical protein TcasGA2_TC030780 | 1.21 |
| comp40980_c0 | - | 1.21 |
| comp75355_c0_orf1 | eukaryotic translation initiation factor 3 subunit G | 1.21 |
| c56074_g1 | - | 1.21 |
| c67794_g1 | - | 1.21 |
| comp74038_c0_orf1 | AGAP011399-PA | 1.21 |
| comp74036_c2_orf1 | PREDICTED: NADH dehydrogenase [ubiquinone] 1 alpha subcomplex subunit 1 | 1.21 |
| comp70830_c1_orf1 | conserved hypothetical protein | 1.21 |
| comp41760_c1_orf1 | GF11465 | 1.21 |
| c59347_g2 | gliotactin, isoform B | 1.21 |
| comp73661_c0_orf1 | PREDICTED: splicing factor 3A subunit 1 | 1.21 |
| c67248_g1 | PREDICTED: hypothetical protein LOC100167398 | 1.21 |
| c64398_g1 | PREDICTED: similar to CG3983 CG3983-PB | 1.21 |
| **Down-regulated** |  |  |
| comp65762_c0_orf1 | PREDICTED: myosin heavy chain, muscle isoform 1 | 0.62 |
| c68821_g2 | ptpla domain protein, putative | 0.7 |
| c53347_g1 | PREDICTED: band 4.1-like protein 5-like | 0.7 |
| comp70000_c0_orf1 | C-factor, putative | 0.72 |
| c61960_g2 | hypothetical protein SINV_08125 | 0.73 |
| c66630_g1 | COE2, partial | 0.73 |
| comp70145_c0_orf1 | phosphatidylethanolamine-binding protein | 0.73 |
| comp59684_c0_orf1 | glutathione S transferase class delta variant 1 | 0.73 |
| comp68395_c0_orf1 | PREDICTED: SEC14-like protein 3-like | 0.73 |
| c67931_g1 | predicted protein | 0.73 |
| comp65894_c0_orf1 | cathepsin B-N | 0.73 |
| comp74117_c0_orf1 | hypothetical protein TcasGA2_TC003706 | 0.74 |
| comp70583_c2_orf1 | PREDICTED: presqualene diphosphate phosphatase-like | 0.74 |
| c69608_g1 | PREDICTED: neurexin-4-like | 0.74 |
| comp72200_c4_orf2 | putative histidine triad protein member | 0.75 |
| comp580790_c0_orf1 | actin | 0.75 |
| c62307_g1 | PREDICTED: myosin heavy chain, muscle isoform 1 | 0.75 |
| c60367_g1 | PREDICTED: trimeric intracellular cation channel type B-A-like | 0.76 |
| c68307_g2 | hypothetical protein TcasGA2_TC009928 | 0.76 |
| c66619_g4 | PREDICTED: similar to CG9723 CG9723-PA | 0.76 |
| comp61171_c0_orf1 | unnamed protein product | 0.76 |
| c67727_g1 | - | 0.77 |
| c11466_g1 | - | 0.77 |
| comp76311_c0_orf1 | PREDICTED: similar to synaptobrevin | 0.77 |
| comp73113_c2_orf1 | tyrosine/tryptophan monooxygenase, putative | 0.78 |
| c70086_g1 | hypothetical protein TcasGA2_TC004221 | 0.78 |
| c65252_g1 | hypothetical protein TcasGA2_TC008304 | 0.78 |
| comp67364_c0_orf1 | PREDICTED: ribosomal protein S6 kinase alpha-3-like isoform 1 | 0.78 |
| comp46864_c1_orf1 | PREDICTED: phosphatidylinositol 4-kinase alpha-like isoform 2 | 0.79 |
| comp72764_c0_orf1 | hypothetical protein TcasGA2_TC014328 | 0.79 |
| comp66853_c0_orf1 | hypothetical protein TcasGA2_TC003145 | 0.79 |
| c42754_g2 | S1 RNA binding domain containing protein | 0.79 |
| comp73123_c0_orf1 | conserved hypothetical protein | 0.79 |
| c50021_g1 | PREDICTED: cytochrome b-c1 complex subunit 9-like | 0.8 |
| c56795_g1 | u6 snRNA-associated sm-like protein lsm2 | 0.8 |
| comp61846_c1_orf1 | cytochrome P450 | 0.8 |
| comp74503_c0_orf1 | hypothetical protein TcasGA2_TC008454 | 0.8 |
| c37613_g1 | vitellogenin | 0.8 |
| comp68399_c0_orf1 | PREDICTED: DNA replication licensing factor mcm7-like isoform 1 | 0.8 |
| c50713_g1 | PREDICTED: 3-ketodihydrosphingosine reductase-like | 0.8 |
| c63618_g1 | - | 0.8 |
| comp72159_c1_orf1 | GF11454 | 0.8 |
| c1613_g1 | PREDICTED: guanine nucleotide-binding protein G(s) subunit alpha-like | 0.8 |
| comp72720_c0_orf1 | PREDICTED: TBC domain-containing protein kinase-like protein-like | 0.81 |
| c58571_g1 | phosphatidylethanolamine-binding protein | 0.81 |
| c63198_g1 | PREDICTED: CCR4-NOT transcription complex subunit 7-like | 0.81 |
| c62278_g1 | Microspherule protein 1 | 0.81 |
| c25679_g1 | putative mitochondrial NADH-ubiquinone oxidoreductase AGGG subunit | 0.81 |
| c60561_g1 | cathepsin B-S | 0.81 |
| comp74008_c0_orf1 | PREDICTED: non-lysosomal glucosylceramidase-like | 0.81 |
| comp68870_c3_orf2 | PREDICTED: COMM domain-containing protein 8 | 0.81 |
| c64030_g1 | - | 0.81 |
| c63526_g1 | hypothetical protein TcasGA2_TC010433 | 0.81 |
| c51011_g1 | ribosomal protein S15Aa, isoform D | 0.81 |
| c62876_g1 | 2-deoxyribose-5-phosphate aldolase homolog (C. elegans) | 0.82 |
| c63168_g2 | PREDICTED: ATP-binding cassette sub-family G member 4-like isoform 1 | 0.82 |
| comp63738_c0_orf1 | tyrosine-protein phosphatase corkscrew, putative | 0.82 |
| c55589_g1 | acyl-CoA-binding protein | 0.82 |
| c56365_g1 | eukaryotic translation initiation factor 3 subunit 12-like protein | 0.82 |
| comp67452_c0_orf1 | PREDICTED: traB domain-containing protein-like isoform 2 | 0.82 |
| comp71710_c0_orf1 | PREDICTED: glucosamine-6-phosphate isomerase isoform 1 | 0.82 |
| comp71804_c0_orf1 | PREDICTED: puromycin-sensitive aminopeptidase-like | 0.82 |
| comp71613_c0_orf1 | PREDICTED: serine/threonine-protein phosphatase 5-like | 0.83 |
| comp63275_c0 | - | 0.83 |
| comp58633_c0_orf1 | PREDICTED: similar to CG8128 CG8128-PA | 0.83 |
| c68792_g2 | PREDICTED: UPF0160 protein MYG1, mitochondrial-like | 0.83 |
| c67690_g2 | hypothetical protein BRAFLDRAFT_66468 | 0.83 |
| comp58745_c0_orf1 | unknown | 0.83 |
| c57778_g1 | hypothetical protein TcasGA2_TC013249 | 0.83 |
| c59635_g1 | hypothetical protein SINV_08840 | 0.83 |
| comp62425_c0 | - | 0.83 |
| comp76071_c0_orf1 | conserved hypothetical protein | 0.83 |
| c62511_g1 | aminopeptidase-like protein | 0.83 |
| c62993_g1 | PREDICTED: hypothetical protein LOC100118865 isoform 1 | 0.83 |
| c62557_g1 | AGAP004533-PA | 0.83 |
| c67051_g1 | COP9 signalosome complex subunit, putative | 0.83 |
| comp41053_c0_orf1 | ribosomal protein L18A | 0.83 |

**Table S3. DEPs identified in the comparison of PaLCuCNV-infected vs. un-infected.**

| **Protein ID** | **Protein name** | **Fold change** |
| --- | --- | --- |
| **Up-regulated** |  |  |
| comp67637_c0_orf1 | PREDICTED: ras-related protein Rap-1b-like isoform 1 | 2.3 |
| c61139_g1 | PREDICTED: protein msta, isoform B-like | 2.16 |
| comp60292_c0_orf1 | TPA_inf: cathepsin B | 2.01 |
| c67729_g1 | PREDICTED: similar to phospholipase A2, group VI (cytosolic, calcium-independent) | 1.96 |
| comp66486_c0_orf1 | PREDICTED: elongation of very long chain fatty acids protein 4-like | 1.95 |
| c64195_g1 | PREDICTED: sodium/calcium exchanger 1-like | 1.85 |
| c64800_g1 | AAEL006918-PB | 1.83 |
| comp74417_c0_orf1 | hypothetical protein TcasGA2_TC004221 | 1.81 |
| c63170_g1 | PREDICTED: vacuolar protein sorting-associated protein 29-like | 1.75 |
| c56768_g1 | actin | 1.73 |
| c38279_g1 | PREDICTED: SH3 domain-binding glutamic acid-rich protein homolog | 1.72 |
| c51657_g1 | AGAP007992-PA | 1.71 |
| comp73512_c0_orf1 | hypothetical protein SINV_15050 | 1.7 |
| c64110_g1 | Splicing factor, arginine/serine-rich 2 | 1.69 |
| c44423_g2 | hypothetical protein DAPPUDRAFT_307391 | 1.67 |
| c153250_g1 | GH22984 | 1.67 |
| c63230_g1 | putative defense protein 3 | 1.66 |
| comp67402_c3_orf2 | D-beta-hydroxybutyrate dehydrogenase, putative | 1.66 |
| comp879424_c0_orf1 | PREDICTED: chaoptin-like | 1.62 |
| c65422_g1 | - | 1.6 |
| comp58993_c0_orf1 | cdk1 | 1.6 |
| c66096_g5 | conserved hypothetical protein | 1.58 |
| comp65705_c0_orf1 | PREDICTED: retinol dehydrogenase 11-like isoform 1 | 1.56 |
| c52295_g1 | GM24606p | 1.56 |
| comp41074_c1_orf1 | PREDICTED: small ubiquitin-related modifier-like | 1.56 |
| comp73324_c0_orf1 | PREDICTED: heterogeneous nuclear ribonucleoprotein K-like isoform 1 | 1.56 |
| c63913_g1 | hypothetical protein KGM_22666 | 1.55 |
| comp71123_c1_orf1 | PREDICTED: similar to AGAP010450-PA | 1.53 |
| c61923_g1 | nuclear protein | 1.53 |
| comp58988_c0_orf1 | AAEL010801-PB | 1.53 |
| c69101_g1 | - | 1.52 |
| comp52386_c0_orf1 | PREDICTED: DNA polymerase delta subunit 2-like | 1.51 |
| c57402_g1 | hypothetical protein KGM_07372 | 1.51 |
| c1880_g1 | PREDICTED: protein LSM12 homolog | 1.51 |
| comp72848_c1_orf1 | conserved hypothetical protein | 1.5 |
| comp29955_c0_orf1 | ribosomal protein L28-like protein | 1.5 |
| c61399_g1 | hypothetical protein KGM_08367 | 1.5 |
| c60892_g1 | - | 1.5 |
| comp72309_c1_orf1 | PREDICTED: similar to rab gdp/gtp exchange factor isoform 1 | 1.5 |
| fig\|572265_7_peg_974 | - | 1.49 |
| WP_014895133_1 | symbionin symS - pea aphid | 1.48 |
| c64336_g1 | nuclear pore complex protein nup154, putative | 1.48 |
| c64603_g2 | hypothetical protein DAPPUDRAFT_42659 | 1.48 |
| comp67813_c0_orf1 | PREDICTED: hypothetical protein LOC100166909 | 1.48 |
| comp69806_c0_orf1 | organic cation transporter protein | 1.48 |
| c61487_g1 | PREDICTED: similar to arginyl-tRNA synthetase | 1.46 |
| c70603_g1 | - | 1.45 |
| c61243_g1 | GI16849 | 1.45 |
| comp477134_c0_orf1 | PREDICTED: hemicentin-1-like isoform 2 | 1.45 |
| c59925_g1 | 39S ribosomal protein L53, mitochondrial | 1.45 |
| c53473_g1 | conserved hypothetical protein | 1.44 |
| comp64934_c0_orf1 | cysteine peptidase isoform b | 1.43 |
| comp69323_c1 | - | 1.43 |
| comp71236_c0_orf1 | PREDICTED: surfeit locus protein 1-like | 1.42 |
| comp63595_c0_orf1 | PREDICTED: hypothetical protein LOC100166785 | 1.42 |
| comp71074_c0_orf1 | nucleolysin TIA-1, putative | 1.41 |
| comp70776_c1_orf1 | PREDICTED: protein alan shepard-like | 1.41 |
| comp380948_c0_orf1 | PREDICTED: laminin subunit alpha-like | 1.41 |
| c35339_g1 | - | 1.41 |
| comp67133_c0_orf1 | PREDICTED: glucose dehydrogenase [acceptor]-like | 1.4 |
| c58916_g1 | PREDICTED: blood vessel epicardial substance-like isoform 1 | 1.4 |
| comp56573_c1_orf1 | PREDICTED: dephospho-CoA kinase domain-containing protein-like | 1.4 |
| c57159_g1 | GJ20513 | 1.4 |
| c16261_g1 | PREDICTED: similar to maggie CG14981-PA | 1.39 |
| c22589_g1 | - | 1.39 |
| c68917_g1 | PREDICTED: rap guanine nucleotide exchange factor 6-like, partial | 1.38 |
| comp60414_c0 | - | 1.38 |
| comp55754_c0_orf1 | conserved hypothetical protein | 1.38 |
| comp64164_c0_orf1 | lysine-specific histone demethylase, putative | 1.38 |
| c64891_g1 | PREDICTED: similar to cuticular protein 127, RR-1 family (AGAP000344-PA) | 1.38 |
| comp73950_c0 | - | 1.37 |
| comp68150_c3_orf1 | AGAP008876-PA | 1.37 |
| c1950_g1 | putative ribosomal protein S25 | 1.37 |
| c69805_g9 | ACYPI38240 | 1.37 |
| c68616_g1 | - | 1.37 |
| comp68485_c0_orf1 | PREDICTED: short-chain dehydrogenase/reductase family 16C member 6-like isoform 1 | 1.37 |
| c61942_g1 | - | 1.37 |
| c61447_g1 | membrane-bound trehalase | 1.37 |
| comp72022_c1_orf1 | PREDICTED: four and a half LIM domains protein 2-like isoform 1 | 1.37 |
| comp70201_c0_orf1 | alkaline phosphatase | 1.36 |
| comp40980_c0 | - | 1.36 |
| c62046_g1 | unnamed protein product | 1.36 |
| comp250437_c0_orf1 | integrin alpha-PS2 precursor, putative | 1.36 |
| comp59163_c0_orf1 | conserved hypothetical protein | 1.36 |
| c30353_g1 | orcokinin | 1.36 |
| comp69758_c2_orf1 | GK25645 | 1.36 |
| c68842_g2 | PREDICTED: similar to groucho protein | 1.36 |
| c66344_g1 | PREDICTED: heterogeneous nuclear ribonucleoprotein L-like | 1.36 |
| comp647510_c0_orf1 | hypothetical protein KGM_12959 | 1.35 |
| comp68977_c1_orf1 | PREDICTED: serine/threonine-protein phosphatase 2B catalytic subunit 3-like | 1.35 |
| comp61375_c0_orf1 | hypothetical protein | 1.35 |
| c56539_g1 | uncharacterized protein LOC100163911 | 1.35 |
| comp73307_c0_orf2 | PREDICTED: uncharacterized protein LOC100882936 | 1.35 |
| comp76302_c0_orf1 | membrane-associated protein, putative | 1.35 |
| c58919_g1 | - | 1.34 |
| c68898_g1 | PREDICTED: protein phosphatase methylesterase 1-like | 1.34 |
| comp73740_c0_orf1 | GK15947 | 1.34 |
| c65726_g1 | PREDICTED: v-type proton ATPase 116 kDa subunit a isoform 1-like | 1.34 |
| c61021_g1 | RNA polymerase-associated protein LEO1, putative | 1.34 |
| c64247_g6 | hypothetical protein DAPPUDRAFT_301871 | 1.34 |
| comp70962_c0_orf1 | flotillin-1 | 1.34 |
| comp70727_c0_orf1 | PREDICTED: rho guanine nucleotide exchange factor 7-like isoform 1 | 1.33 |
| c59202_g1 | unknown | 1.33 |
| comp69879_c0_orf2 | adapter molecule Crk | 1.33 |
| c56034_g1 | PREDICTED: hypothetical protein LOC409240 | 1.33 |
| c67538_g1 | methionine sulfoxide reductase B3 isoform 1 | 1.33 |
| comp74180_c0_orf1 | PREDICTED: similar to AGAP010237-PA | 1.33 |
| comp65193_c0_orf1 | PREDICTED: similar to Sel1l protein | 1.33 |
| c121590_g1 | hypothetical protein TcasGA2_TC012992 | 1.33 |
| comp72185_c0_orf1 | PREDICTED: prostatic acid phosphatase-like | 1.33 |
| c64398_g1 | PREDICTED: similar to CG3983 CG3983-PB | 1.33 |
| c70930_g4 | - | 1.32 |
| comp40938_c0_orf1 | putative mitochondrial NADH dehydrogenase (ubiquinone) 1 alpha subcomplex | 1.32 |
| comp64445_c0_orf1 | CG12163, isoform B | 1.31 |
| comp73164_c0_orf1 | PREDICTED: phosphatidylethanolamine-binding protein homolog F40A3.3-like isoform 1 | 1.31 |
| c62186_g1 | serine protease P150 | 1.31 |
| comp65735_c0_orf1 | Apoptosis regulator BAX | 1.31 |
| comp59031_c0_orf1 | PREDICTED: similar to predicted protein | 1.31 |
| comp66575_c0_orf1 | PREDICTED: ATP-binding cassette sub-family G member 1-like | 1.3 |
| comp67862_c0_orf1 | PREDICTED: UPF0451 protein C17orf61 homolog | 1.3 |
| comp65478_c0_orf1 | PREDICTED: clathrin light chain-like | 1.3 |
| comp61231_c0_orf1 | PREDICTED: hypothetical protein LOC100647906 | 1.3 |
| c25716_g1 | low-density lipoprotein receptor, putative | 1.3 |
| c143871_g1 | PREDICTED: muscle M-line assembly protein unc-89-like | 1.3 |
| c165725_g1 | cathepsin B | 1.3 |
| comp65648_c0_orf1 | hypothetical protein TcasGA2_TC004143 | 1.3 |
| comp52231_c1_orf1 | putative 60S ribosomal protein L13a | 1.3 |
| comp54332_c0 | - | 1.29 |
| comp71764_c0_orf1 | PREDICTED: thiamin pyrophosphokinase 1-like | 1.29 |
| c59346_g1 | actin [Ornithodoros moubata] | 1.29 |
| comp59039_c0 | - | 1.29 |
| comp67502_c0_orf1 | PREDICTED: UDP-glucuronosyltransferase 1-7-like | 1.29 |
| c59013_g1 | PREDICTED: protein jagunal-like | 1.29 |
| comp73100_c2_orf1 | GJ11709 | 1.29 |
| comp56451_c0_orf1 | PREDICTED: 60S ribosomal protein L7a-like | 1.28 |
| comp68746_c0_orf1 | hypothetical protein TcasGA2_TC030780 | 1.28 |
| c59908_g2 | PREDICTED: similar to AGAP008909-PA | 1.28 |
| comp70003_c0_orf1 | PREDICTED: CLIP-associating protein 1-like | 1.28 |
| c51220_g1 | PREDICTED: icarapin-like | 1.28 |
| comp29968_c0_orf1 | PREDICTED: hypothetical protein LOC100746188 | 1.28 |
| c3648_g1 | PREDICTED: similar to ATP synthase delta chain, mitochondrial | 1.28 |
| c65822_g1 | - | 1.28 |
| comp67677_c0_orf1 | phosphatidylethanolamine-binding protein | 1.28 |
| comp40702_c0_orf1 | hypothetical protein DAPPUDRAFT_110732 | 1.28 |
| comp70483_c0_orf1 | PREDICTED: similar to CG5758 CG5758-PA | 1.27 |
| c43129_g1 | DPY-30 protein, putative | 1.27 |
| comp63139_c0_orf1 | 60S ribosomal protein L8-like | 1.27 |
| c48270_g1 | ACYPI008219 | 1.27 |
| comp66975_c0 | - | 1.27 |
| comp70976_c0_orf1 | lanosterol synthase | 1.27 |
| c57283_g1 | translation elongation factor EF-1 alpha/Tu | 1.27 |
| comp75481_c0_orf1 | unknown | 1.27 |
| comp39853_c0 | - | 1.26 |
| comp69527_c0 | - | 1.26 |
| comp56975_c0_orf1 | histone H2AV | 1.26 |
| c69852_g2 | sjoegren syndrome nuclear autoantigen 1-like protein | 1.26 |
| c54979_g1 | C-terminal-binding protein | 1.26 |
| comp72119_c0_orf1 | stromal interaction molecule-like protein | 1.26 |
| comp59007_c0_orf1 | PREDICTED: ankyrin repeat and LEM domain-containing protein 2-like | 1.26 |
| comp69919_c0_orf1 | GI13082 | 1.26 |
| comp87167_c0_orf1 | hypothetical protein Phum_PHUM213210 | 1.26 |
| comp74818_c0_orf1 | cd36 antigen | 1.26 |
| c59347_g2 | gliotactin, isoform B | 1.26 |
| c69034_g2 | PREDICTED: similar to AGAP006340-PC | 1.26 |
| c63762_g1 | PREDICTED: hypothetical protein LOC100162711 | 1.25 |
| c68324_g2 | unknown | 1.25 |
| c59487_g1 | PREDICTED: hypothetical protein LOC100570329 | 1.25 |
| comp71783_c1_orf1 | putative chemosensory protein CSP6 | 1.25 |
| c182532_g1 | GMP synthetase, putative | 1.25 |
| comp74664_c0_orf1 | PREDICTED: ras-related protein Rap-2c-like | 1.25 |
| comp62073_c0_orf1 | PREDICTED: exocyst complex component 6B-like isoform 2 | 1.25 |
| WP_014895142_1 | - | 1.25 |
| comp61957_c0 | - | 1.25 |
| c44482_g1 | PREDICTED: proteasome subunit alpha type-3-like | 1.25 |
| comp64227_c0_orf1 | PREDICTED: similar to reticulocalbin | 1.25 |
| c70349_g1 | cysteine string protein | 1.25 |
| comp61331_c0_orf1 | PREDICTED: L-lactate dehydrogenase-like | 1.25 |
| c44015_g1 | PREDICTED: tropomyosin-2-like isoform 2 | 1.25 |
| c57836_g3 | PREDICTED: hypothetical protein LOC100744819 | 1.25 |
| comp70552_c0_orf1 | PREDICTED: transaldolase-like | 1.24 |
| c59173_g1 | PREDICTED: hypothetical protein LOC100165581 | 1.24 |
| comp70406_c0_orf1 | PREDICTED: similar to DEAD-box RNA-dependent helicase p68 | 1.24 |
| comp74497_c0_orf1 | tdrd7 | 1.24 |
| c62440_g1 | - | 1.24 |
| comp75391_c0_orf1 | PREDICTED: proteasome subunit alpha type-4-like | 1.24 |
| c62422_g1 | PREDICTED: n-acetyl-D-glucosamine kinase-like isoform 1 | 1.24 |
| c70690_g1 | argonaute-2 | 1.24 |
| c56074_g1 | - | 1.24 |
| comp303744_c0 | - | 1.24 |
| comp29923_c0_orf1 | PREDICTED: similar to GA20229-PA | 1.24 |
| c64649_g2 | innexin | 1.24 |
| c64886_g1 | PREDICTED: hypothetical protein LOC100645428 | 1.24 |
| comp73793_c0_orf1 | AGAP003371-PA | 1.24 |
| c67078_g1 | - | 1.24 |
| c65910_g1 | PREDICTED: pumilio homolog 2-like isoform 1 | 1.24 |
| comp59451_c0_orf1 | PREDICTED: G-protein coupled receptor 143-like | 1.24 |
| c67248_g1 | PREDICTED: hypothetical protein LOC100167398 | 1.24 |
| c65285_g4 | PREDICTED: mitochondrial folate transporter/carrier-like | 1.24 |
| c66277_g2 | cathepsin B-5880 precursor | 1.24 |
| comp53394_c0_orf1 | NADH dehydrogenase [ubiquinone] 1 alpha subcomplex subunit 11 | 1.24 |
| comp73790_c0_orf1 | phosphatidylinositol-4-phosphate 5-kinase type-1 alpha | 1.24 |
| c70499_g1 | DD5 | 1.23 |
| c66715_g2 | basigin precursor, putative | 1.23 |
| c62328_g1 | hypothetical protein DAPPUDRAFT_329664 | 1.23 |
| c49442_g1 | putative ATP synthase oligomycin sensitivity conferral protein | 1.23 |
| comp73879_c0_orf1 | PREDICTED: transient receptor potential cation channel protein painless-like isoform 1 | 1.23 |
| comp74141_c2_orf1 | ACYPI003875 | 1.23 |
| comp59266_c0_orf1 | PREDICTED: galactokinase-like | 1.23 |
| c70870_g1 | phosphatidylethanolamine-binding protein | 1.23 |
| comp65802_c0_orf1 | PREDICTED: similar to succinate semialdehyde dehydrogenase, mitochondrial | 1.23 |
| comp52413_c0_orf1 | chickadee | 1.23 |
| comp70509_c0_orf1 | PREDICTED: hypothetical protein LOC100167736 | 1.23 |
| c56033_g1 | PREDICTED: similar to metaxin 2 | 1.23 |
| c67154_g1 | PREDICTED: replication factor C subunit 5-like | 1.23 |
| c60978_g1 | PREDICTED: u4/U6.U5 tri-snRNP-associated protein 2-like | 1.23 |
| c50242_g1 | PREDICTED: hypothetical protein LOC100161256 | 1.23 |
| c70259_g2 | PREDICTED: septin-4-like | 1.23 |
| c25195_g1 | AAEL001615-PA | 1.23 |
| comp74268_c0_orf1 | PREDICTED: phosphatidylethanolamine-binding protein homolog F40A3.3-like isoform 2 | 1.23 |
| c67481_g1 | AGAP009234-PA | 1.23 |
| comp69314_c0_orf2 | PREDICTED: hypothetical protein LOC100572493 | 1.22 |
| c66419_g1 | arginine/serine-rich splicing factor, putative | 1.22 |
| comp64613_c0_orf1 | takeout/JHBP like protein | 1.22 |
| comp53515_c0_orf1 | PREDICTED: similar to phosphoacetylglucosamine mutase | 1.22 |
| comp56996_c0_orf1 | AGAP006179-PB | 1.22 |
| c66598_g1 | hypothetical protein TcasGA2_TC007283 | 1.22 |
| c55942_g1 | PREDICTED: proteasomal ubiquitin receptor ADRM1-like | 1.22 |
| c61881_g1 | PREDICTED: acetylserotonin O-methyltransferase-like | 1.22 |
| c62318_g1 | hypothetical protein KGM_10424 | 1.22 |
| comp70499_c1_orf1 | GF24985 | 1.22 |
| comp56704_c0_orf1 | PREDICTED: peptidyl-prolyl cis-trans isomerase B-like | 1.22 |
| c45814_g1 | juvenile hormone binding protein | 1.22 |
| comp71497_c1_orf1 | PREDICTED: cAMP-dependent protein kinase catalytic subunit-like | 1.22 |
| comp74212_c0_orf1 | PREDICTED: protein retinal degeneration B-like | 1.21 |
| comp67785_c0_orf1 | replication protein A 70 kDa DNA-binding subunit | 1.21 |
| c51152_g1 | DEAD box ATP-dependent RNA helicase | 1.21 |
| c59207_g2 | - | 1.21 |
| comp65336_c0_orf1 | - | 1.21 |
| c60853_g1 | hemocyte aggregation inhibitor protein precursor | 1.21 |
| comp69458_c0_orf1 | gamma-soluble NSF attachment protein, putative | 1.21 |
| c62324_g1 | PREDICTED: zinc finger protein 161 homolog | 1.21 |
| c67854_g1 | PREDICTED: similar to CG3662 CG3662-PA | 1.21 |
| comp74934_c0_orf1 | PREDICTED: hypothetical protein LOC100168823 | 1.21 |
| comp39103_c1_orf1 | fasciclin-1 | 1.21 |
| c67573_g1 | - | 1.21 |
| comp45791_c0_orf1 | cuticular protein | 1.21 |
| comp69511_c0_orf1 | PREDICTED: transcriptional regulator ATRX homolog | 1.21 |
| comp73479_c0_orf1 | vacuolar ATP synthase subunit S1, putative | 1.21 |
| c66686_g2 | - | 1.21 |
| c55056_g1 | PREDICTED: similar to XPA-binding protein 2 | 1.21 |
| comp74041_c0_orf1 | RNA polymerase II second largest subunit | 1.21 |
| c58687_g1 | PREDICTED: nucleoporin p58/p45 | 1.21 |
| comp66291_c0_orf1 | import inner membrane translocase subunit TIM50-C, putative | 1.21 |
| c65657_g2 | PREDICTED: hypothetical protein LOC724126 | 1.21 |
| c66786_g2 | Thyroid peroxidase precursor, putative | 1.21 |
| c60036_g1 | FAD oxidoreductase | 1.21 |
| c64864_g1 | PREDICTED: retinol dehydrogenase 14-like | 1.21 |
| comp73638_c1_orf1 | hypothetical protein DAPPUDRAFT_311676 | 1.21 |
| c70943_g2 | transmembrane protein 62 | 1.21 |
| comp73661_c0_orf1 | PREDICTED: splicing factor 3A subunit 1 | 1.21 |
| comp61973_c0_orf1 | dual specificity protein phosphatase CDC14A | 1.21 |
| c36035_g1 | - | 1.21 |
| c59927_g1 | PREDICTED: probable enoyl-CoA hydratase, mitochondrial-like | 1.21 |
| c32538_g1 | - | 1.21 |
| comp70989_c0_orf1 | PREDICTED: hypothetical protein LOC100748451 | 1.21 |
| **Down-regulated** |  |  |
| comp55351_c0_orf1 | actin type 2 | 0.34 |
| comp1063366_c0_orf1 | ADP/ATP-carrier protein, partial | 0.45 |
| comp72200_c4_orf2 | putative histidine triad protein member | 0.63 |
| c63442_g1 | PREDICTED: ADP-ribosylation factor-like protein 1-like isoform 1 | 0.65 |
| c68307_g2 | hypothetical protein TcasGA2_TC009928 | 0.66 |
| c68060_g1 | PREDICTED: hypothetical protein LOC100164562 | 0.69 |
| c69608_g1 | PREDICTED: neurexin-4-like | 0.69 |
| comp68396_c0_orf1 | hypothetical protein TcasGA2_TC005739 | 0.7 |
| c68821_g2 | ptpla domain protein, putative | 0.7 |
| c62307_g1 | PREDICTED: myosin heavy chain, muscle isoform 1 | 0.7 |
| comp65762_c0_orf1 | PREDICTED: myosin heavy chain, muscle isoform 1 | 0.71 |
| comp70145_c0_orf1 | phosphatidylethanolamine-binding protein | 0.71 |
| comp70602_c0_orf1 | PREDICTED: ankyrin repeat domain-containing protein 13C-like | 0.72 |
| comp61171_c0_orf1 | unnamed protein product | 0.72 |
| c53347_g1 | PREDICTED: band 4.1-like protein 5-like | 0.72 |
| c56365_g1 | eukaryotic translation initiation factor 3 subunit 12-like protein | 0.73 |
| c63198_g1 | PREDICTED: CCR4-NOT transcription complex subunit 7-like | 0.73 |
| c65809_g1 | PREDICTED: cullin-3-A-like | 0.73 |
| c11466_g1 | - | 0.73 |
| c63800_g2 | lysosomal acid phosphatase precursor, putative | 0.74 |
| comp29924_c0_orf1 | hypothetical protein CAPTEDRAFT_50237, partial | 0.74 |
| comp70583_c2_orf1 | PREDICTED: presqualene diphosphate phosphatase-like | 0.75 |
| c65252_g1 | hypothetical protein TcasGA2_TC008304 | 0.75 |
| c50713_g1 | PREDICTED: 3-ketodihydrosphingosine reductase-like | 0.75 |
| c73842_g1 | PREDICTED: hypothetical protein LOC100568595 isoform 1 | 0.75 |
| comp72159_c1_orf1 | GF11454 | 0.75 |
| c67690_g2 | hypothetical protein BRAFLDRAFT_66468 | 0.76 |
| c57778_g1 | hypothetical protein TcasGA2_TC013249 | 0.76 |
| comp74297_c0_orf1 | PREDICTED: similar to membrin | 0.76 |
| c50021_g1 | PREDICTED: cytochrome b-c1 complex subunit 9-like | 0.77 |
| c58929_g2 | PREDICTED: ras-related protein Rab-39B-like | 0.77 |
| c63168_g2 | PREDICTED: ATP-binding cassette sub-family G member 4-like isoform 1 | 0.77 |
| c60020_g1 | serine/threonine-protein phosphatase alpha-1 isoform | 0.77 |
| comp72495_c0_orf1 | PREDICTED: probable maltase L-like | 0.77 |
| comp68321_c0_orf1 | male sterility domain-containing protein, putative | 0.77 |
| c61534_g1 | GF16442 | 0.77 |
| c58571_g1 | phosphatidylethanolamine-binding protein | 0.78 |
| comp70166_c0 | - | 0.78 |
| c59635_g1 | hypothetical protein SINV_08840 | 0.78 |
| comp59684_c0_orf1 | glutathione S transferase class delta variant 1 | 0.78 |
| c66083_g1 | PREDICTED: congested-like trachea protein-like | 0.78 |
| c15820_g1 | predicted protein | 0.78 |
| WP_014894974_1 | hypothetical protein CAPTEDRAFT_97159, partial | 0.78 |
| comp65316_c0_orf1 | N-acetyltransferase-like | 0.78 |
| comp4183_c0_orf1 | predicted protein | 0.78 |
| comp74336_c0_orf1 | PREDICTED: c-myc promoter-binding protein-like isoform 2 | 0.78 |
| WP_014895007_1 | PREDICTED: 30S ribosomal protein S5-like, partial | 0.79 |
| c68819_g1 | sucrase | 0.79 |
| c48683_g1 | hypothetical protein DAPPUDRAFT_332854 | 0.79 |
| c6484_g1 | PREDICTED: probable ATP-dependent RNA helicase DHX36-like | 0.79 |
| c70620_g2 | hypothetical protein TcasGA2_TC009928 | 0.79 |
| comp68198_c0_orf1 | hydroxymethylglutaryl-CoA synthase 1 | 0.79 |
| c182801_g1 | PREDICTED: nipped-B-like protein | 0.79 |
| c62963_g1 | PREDICTED: twinkle protein, mitochondrial-like | 0.79 |
| c66721_g6 | PREDICTED: acyl-CoA:lysophosphatidylglycerol acyltransferase 1-like isoform 1 | 0.79 |
| c42656_g1 | 14-3-3 zeta | 0.79 |
| c48872_g1 | predicted protein | 0.79 |
| comp29864_c0 | - | 0.8 |
| comp71603_c2_orf1 | PREDICTED: u3 small nucleolar RNA-associated protein 6 homolog | 0.8 |
| comp71500_c0_orf2 | PREDICTED: similar to AGAP004772-PA | 0.8 |
| comp74503_c0_orf1 | hypothetical protein TcasGA2_TC008454 | 0.8 |
| c60302_g1 | PREDICTED: hypothetical protein LOC100161931 | 0.8 |
| c69473_g7 | hypothetical protein KGM_15082 | 0.8 |
| WP_014975147_1 | 2-oxoglutarate dehydrogenase, E2 component, dihydrolipoamide succinyltransferase family protein | 0.8 |
| c63706_g1 | hypothetical protein KGM_22037 | 0.8 |
| comp74690_c0_orf1 | hypothetical protein TcasGA2_TC003675 | 0.8 |
| c56868_g1 | AAEL006847-PA | 0.8 |
| comp70000_c0_orf1 | C-factor, putative | 0.8 |
| comp70633_c0_orf1 | PREDICTED: phosphatidylethanolamine-binding protein homolog F40A3.3-like isoform 1 | 0.8 |
| comp70158_c0_orf1 | PREDICTED: cryptochrome-1-like | 0.8 |
| c63526_g1 | hypothetical protein TcasGA2_TC010433 | 0.8 |
| c64619_g1 | AGAP005490-PA | 0.8 |
| c65561_g2 | ras-related protein Rab-21 | 0.81 |
| comp75854_c0_orf1 | wd-repeat protein | 0.81 |
| comp46864_c1_orf1 | PREDICTED: phosphatidylinositol 4-kinase alpha-like isoform 2 | 0.81 |
| comp58269_c0_orf1 | PREDICTED: 5-formyltetrahydrofolate cyclo-ligase-like | 0.81 |
| c66630_g1 | COE2, partial | 0.81 |
| c51005_g1 | GM13182 | 0.81 |
| c62825_g1 | nicotinamide riboside kinase 1 | 0.81 |
| c63033_g2 | PREDICTED: LOW QUALITY PROTEIN: JNK-interacting protein 3-like | 0.81 |
| comp64520_c1_orf1 | predicted protein | 0.81 |
| comp62425_c0 | - | 0.81 |
| comp68395_c0_orf1 | PREDICTED: SEC14-like protein 3-like | 0.81 |
| c49542_g1 | enolase 3 | 0.81 |
| c64645_g1 | PREDICTED: CCR4-NOT transcription complex subunit 1 isoform 1 | 0.81 |
| c69370_g1 | PREDICTED: barrier-to-autointegration factor-like | 0.81 |
| c36654_g1 | PREDICTED: UPF0545 protein C22orf39 homolog | 0.81 |
| c68226_g2 | PREDICTED: similar to AGAP001629-PA | 0.81 |
| c62530_g1 | PREDICTED: 39S ribosomal protein L35, mitochondrial-like | 0.81 |
| c51011_g1 | ribosomal protein S15Aa, isoform D | 0.81 |
| c70031_g3 | hypothetical protein SINV_04921 | 0.82 |
| c67933_g1 | double-stranded RNA-specific editase Adar | 0.82 |
| c67753_g1 | PREDICTED: venom protease-like | 0.82 |
| c67727_g1 | - | 0.82 |
| comp68540_c0_orf1 | PREDICTED: similar to AGAP010217-PA | 0.82 |
| c68792_g2 | PREDICTED: UPF0160 protein MYG1, mitochondrial-like | 0.82 |
| comp74956_c0_orf1 | PREDICTED: nuclear pore membrane glycoprotein 210-like | 0.82 |
| c57846_g1 | - | 0.82 |
| comp76071_c0_orf1 | conserved hypothetical protein | 0.82 |
| c66619_g4 | PREDICTED: similar to CG9723 CG9723-PA | 0.82 |
| comp57981_c0_orf1 | PREDICTED: probable ATP-dependent RNA helicase kurz-like | 0.82 |
| comp72004_c0_orf1 | alpha tocopherol transfer protein | 0.82 |
| comp63036_c0_orf1 | hypothetical protein SINV_07726 | 0.82 |
| c63669_g1 | - | 0.82 |
| comp66616_c0_orf1 | WD repeat domain phosphoinositide-interacting protein 3 | 0.82 |
| comp67726_c0_orf1 | PREDICTED: cell division cycle protein 23 homolog | 0.83 |
| c67391_g1 | PREDICTED: mesencephalic astrocyte-derived neurotrophic factor homolog | 0.83 |
| comp74768_c0_orf1 | PREDICTED: sodium-independent sulfate anion transporter-like | 0.83 |
| comp72876_c0_orf1 | PREDICTED: similar to AGAP004271-PA | 0.83 |
| comp56726_c0_orf1 | Probable RNA helicase armi | 0.83 |
| comp63275_c0 | - | 0.83 |
| c67958_g1 | PREDICTED: cysteinyl-tRNA synthetase, cytoplasmic-like | 0.83 |
| c20911_g1 | chaperonin containing TCP1 | 0.83 |
| comp67937_c0_orf1 | aminopeptidase-like protein | 0.83 |
| c68529_g2 | - | 0.83 |
| c49494_g2 | trehalose 6-phosphate synthase | 0.83 |
| c44954_g1 | PREDICTED: similar to nicotinamide mononucleotide adenylyltransferase 1 | 0.83 |
| comp70328_c0_orf1 | Density-regulated protein | 0.83 |
| comp72307_c0_orf1 | E3 ubiquitin-protein ligase RNF8 | 0.83 |
| comp64425_c1_orf1 | vitellogenin | 0.83 |
| comp41131_c0_orf1 | uncharacterized protein LOC100161110 | 0.83 |
| c50023_g1 | putative mitochondrial ribosomal protein, L37 | 0.83 |
| c46360_g1 | PREDICTED: hypothetical protein LOC100161842 | 0.83 |
| c63618_g1 | - | 0.83 |
| c68972_g2 | PREDICTED: succinate-semialdehyde dehydrogenase, mitochondrial-like isoform 1 | 0.83 |
| comp580790_c0_orf1 | actin | 0.83 |
| c61767_g1 | PREDICTED: protein-tyrosine phosphatase mitochondrial 1-like protein-like | 0.83 |
| comp75277_c0_orf1 | uncharacterized protein LOC100165200 | 0.83 |
| c64033_g2 | aminoacyl tRNA synthase complex-interacting multifunctional protein 1 | 0.83 |
| comp69173_c0_orf1 | histone H3.3 | 0.83 |

**Table S4. DEPs identified in the comparison of TYLCV-infected vs. PaLCuCNV-infected.**

| **Protein ID** | **Protein name** | **Fold change** |
| --- | --- | --- |
| **Up-regulated** |  |  |
| comp55351_c0_orf1 | actin type 2 | 2.75 |
| comp1063366_c0_orf1 | ADP/ATP-carrier protein, partial | 2.6 |
| c63669_g1 | - | 1.49 |
| c49925_g1 | PREDICTED: mediator of RNA polymerase II transcription subunit 18-like | 1.47 |
| c63033_g2 | PREDICTED: LOW QUALITY PROTEIN: JNK-interacting protein 3-like | 1.33 |
| comp29924_c0_orf1 | hypothetical protein CAPTEDRAFT_50237, partial | 1.33 |
| c42916_g1 | transcription factor btf3, putative | 1.31 |
| comp41484_c0_orf1 | PREDICTED: probable uridine-cytidine kinase-like isoform 2 | 1.3 |
| comp73512_c0_orf1 | hypothetical protein SINV_15050 | 1.3 |
| comp74297_c0_orf1 | PREDICTED: similar to membrin | 1.3 |
| c68868_g2 | - | 1.29 |
| comp61327_c0_orf1 | Probable ubiquitin carboxyl-terminal hydrolase FAF-X | 1.27 |
| comp72049_c0_orf1 | PREDICTED: putative serine protease K12H4.7-like | 1.27 |
| c66072_g1 | PREDICTED: RNA 3'-terminal phosphate cyclase-like protein-like | 1.27 |
| c70031_g3 | hypothetical protein SINV_04921 | 1.25 |
| c67933_g1 | double-stranded RNA-specific editase Adar | 1.25 |
| c121343_g1 | - | 1.25 |
| c69505_g1 | PREDICTED: conserved oligomeric Golgi complex subunit 5-like | 1.25 |
| comp67338_c1_orf1 | UBX domain-containing protein 1 | 1.25 |
| c65325_g2 | - | 1.24 |
| comp74768_c0_orf1 | PREDICTED: sodium-independent sulfate anion transporter-like | 1.24 |
| comp41022_c0_orf1 | vitellogenin-1 | 1.24 |
| c72114_g1 | predicted protein | 1.24 |
| comp68396_c0_orf1 | hypothetical protein TcasGA2_TC005739 | 1.23 |
| comp69033_c2_orf1 | hexamerin 1 | 1.23 |
| comp68198_c0_orf1 | hydroxymethylglutaryl-CoA synthase 1 | 1.23 |
| c66407_g1 | U1 small nuclear ribonucleoprotein 70 kDa | 1.23 |
| c55481_g1 | armadillo protein | 1.23 |
| comp68540_c0_orf1 | PREDICTED: similar to AGAP010217-PA | 1.22 |
| c182801_g1 | PREDICTED: nipped-B-like protein | 1.22 |
| comp73656_c0_orf1 | PREDICTED: acetyl-CoA carboxylase-like isoform 1 | 1.22 |
| c63201_g1 | PREDICTED: WD40 repeat-containing protein SMU1-like isoform 1 | 1.22 |
| comp70158_c0_orf1 | PREDICTED: cryptochrome-1-like | 1.22 |
| comp60560_c0_orf1 | COP9 signalosome complex subunit, putative | 1.21 |
| comp64370_c0_orf1 | PREDICTED: hypothetical protein LOC100573127 | 1.21 |
| c62081_g1 | PREDICTED: uncharacterized protein C9E9.15-like | 1.21 |
| **Down-regulated** |  |  |
| c61139_g1 | PREDICTED: protein msta, isoform B-like | 0.51 |
| c56768_g1 | actin | 0.56 |
| c44423_g2 | hypothetical protein DAPPUDRAFT_307391 | 0.64 |
| c61960_g2 | hypothetical protein SINV_08125 | 0.65 |
| comp71123_c1_orf1 | PREDICTED: similar to AGAP010450-PA | 0.71 |
| c66619_g3 | PREDICTED: polypyrimidine tract-binding protein 1-like isoform 2 | 0.73 |
| comp70201_c0_orf1 | alkaline phosphatase | 0.75 |
| c61399_g1 | hypothetical protein KGM_08367 | 0.75 |
| comp72649_c0_orf1 | PREDICTED: hypothetical protein LOC100114451 | 0.75 |
| comp61331_c0_orf1 | PREDICTED: L-lactate dehydrogenase-like | 0.75 |
| comp65648_c0_orf1 | hypothetical protein TcasGA2_TC004143 | 0.76 |
| comp69758_c2_orf1 | GK25645 | 0.77 |
| comp69806_c0_orf1 | organic cation transporter protein | 0.77 |
| comp71459_c0_orf1 | peroxiredoxin-like protein | 0.78 |
| c58687_g1 | PREDICTED: nucleoporin p58/p45 | 0.78 |
| comp72022_c1_orf1 | PREDICTED: four and a half LIM domains protein 2-like isoform 1 | 0.79 |
| comp73307_c0_orf2 | PREDICTED: uncharacterized protein LOC100882936 | 0.8 |
| comp41725_c0_orf1 | PREDICTED: CDP-diacylglycerol--glycerol-3-phosphate 3-phosphatidyltransferase, mitochondrial-like | 0.8 |
| c70086_g1 | hypothetical protein TcasGA2_TC004221 | 0.81 |
| c59173_g1 | PREDICTED: hypothetical protein LOC100165581 | 0.81 |
| c55687_g1 | protein farnesyltransferase/geranylgeranyltransferase type-1 subunit alpha | 0.81 |
| comp68870_c3_orf2 | PREDICTED: COMM domain-containing protein 8 | 0.81 |
| comp65464_c0_orf1 | leucine carboxyl methyltransferase | 0.81 |
| comp64934_c0_orf1 | cysteine peptidase isoform b | 0.82 |
| comp60292_c0_orf1 | cathepsin B | 0.82 |
| c61487_g1 | PREDICTED: similar to arginyl-tRNA synthetase | 0.82 |
| comp71870_c0_orf1 | PREDICTED: similar to sulfate transporter | 0.82 |
| c62273_g1 | PREDICTED: dymeclin-like | 0.82 |
| comp67904_c0_orf1 | leucine-rich repeat-containing protein 57 | 0.83 |
| comp65585_c0_orf1 | PREDICTED: hypothetical protein | 0.83 |
| WP_014895153_1 | PREDICTED: 30S ribosomal protein S9-like | 0.83 |
| c53473_g1 | conserved hypothetical protein | 0.83 |
| c70690_g1 | argonaute-2 | 0.83 |
| comp67364_c0_orf1 | PREDICTED: ribosomal protein S6 kinase alpha-3-like isoform 1 | 0.83 |
| comp76311_c0_orf1 | PREDICTED: similar to synaptobrevin | 0.83 |
| c60892_g1 | - | 0.83 |
| comp66750_c0_orf1 | PREDICTED: dolichyl-diphosphooligosaccharide--protein glycosyltransferase 48 kDa subunit-like | 0.83 |
| c50035_g1 | PREDICTED: alpha-tocopherol transfer protein-like | 0.83 |
