# Supplementary figures and images for: Comparative proteomic analysis provides new insight into differential transmission of two begomoviruses by a whitefly
Source: Virol J. 2019 Mar 11;16:32. doi: 10.1186/s12985-019-1138-4 (PMC6413443; doi:10.1186/s12985-019-1138-4)

A

TYLCV vs. un-infected

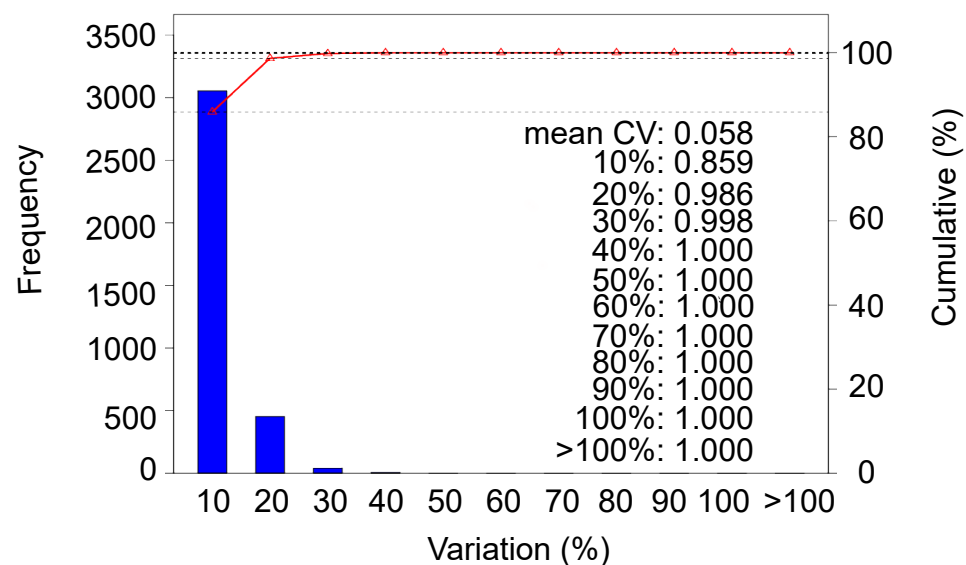

B

PaLCuCNV vs. un-infected

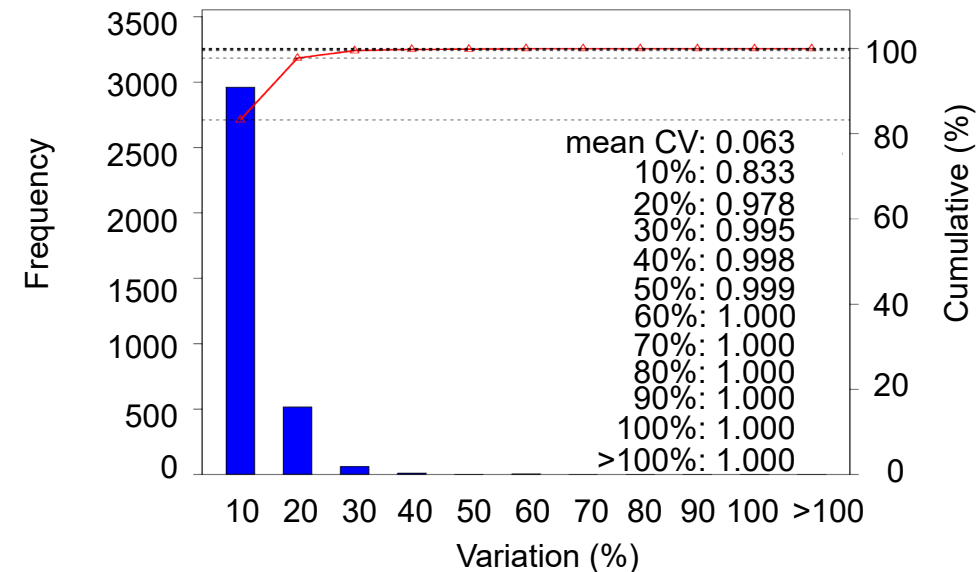

C

TYLCV vs. PaLCuCNV

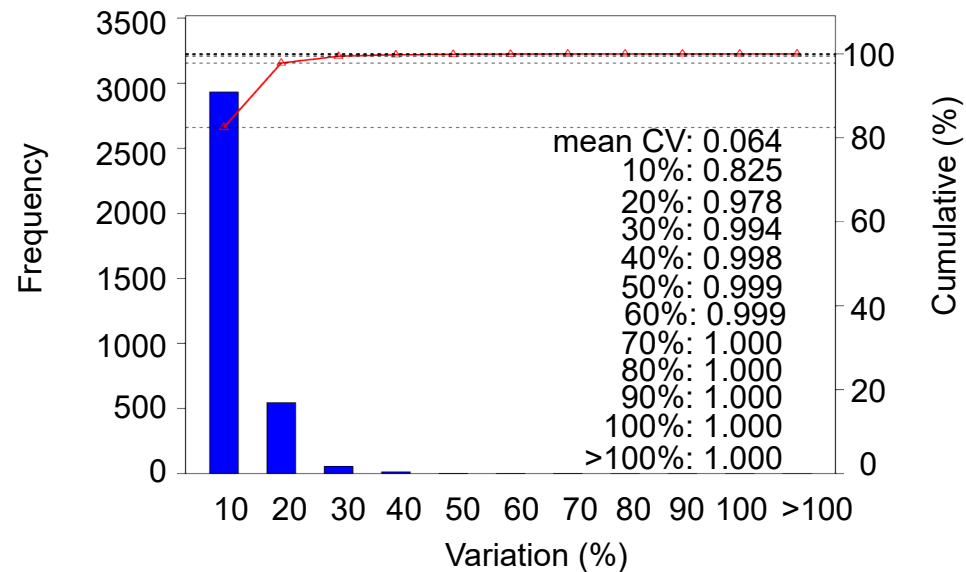

Supplement: Supplementary file 2 — Figure S1. CV distribution of replicates in each of the three combinations for comparison. X-axis is the deviation between the protein ratio of the repeated samples. Y-axis is the percentage of proteins at a certain angle with given levels of quantified proteins. CV value = SD/mean, the lower the value, the better the replication. (A) CV distributions in the comparison of TYLCV-infected vs. un-infected, (B) CV distributions in the comparison of PaLCuCNV-infected vs. un-infected, (C) CV distributions in the comparison of TYLCV-infected vs. PaLCuCNV-infected. (PDF 199 kb) [file 12985_2019_1138_MOESM2_ESM.pdf]
